# Ensemble-based genomic prediction for maize flowering time improves prediction accuracy and reveals novel insights into trait genetic variation

**DOI:** 10.1093/g3journal/jkag090

**Published:** 2026-04-03

**Authors:** Shunichiro Tomura, Owen Powell, Melanie J Wilkinson, Mark Cooper

**Affiliations:** The Queensland Alliance for Agriculture and Food Innovation (QAAFI), Centre for Crop Science, The University of Queensland, St Lucia, QLD 4072, Australia; ARC Centre of Excellence for Plant Success in Nature and Agriculture, The University of Queensland, St Lucia, QLD 4072, Australia; The Queensland Alliance for Agriculture and Food Innovation (QAAFI), Centre for Crop Science, The University of Queensland, St Lucia, QLD 4072, Australia; ARC Centre of Excellence for Plant Success in Nature and Agriculture, The University of Queensland, St Lucia, QLD 4072, Australia; ARC Training Centre in Predictive Breeding, The University of Queensland, St Lucia, QLD 4072, Australia; The Queensland Alliance for Agriculture and Food Innovation (QAAFI), Centre for Crop Science, The University of Queensland, St Lucia, QLD 4072, Australia; ARC Centre of Excellence for Plant Success in Nature and Agriculture, The University of Queensland, St Lucia, QLD 4072, Australia; ARC Training Centre in Predictive Breeding, The University of Queensland, St Lucia, QLD 4072, Australia; The Queensland Alliance for Agriculture and Food Innovation (QAAFI), Centre for Crop Science, The University of Queensland, St Lucia, QLD 4072, Australia; ARC Centre of Excellence for Plant Success in Nature and Agriculture, The University of Queensland, St Lucia, QLD 4072, Australia; ARC Training Centre in Predictive Breeding, The University of Queensland, St Lucia, QLD 4072, Australia

**Keywords:** genomic prediction, ensemble, maize, flowering time, genetic variation

## Abstract

While various genomic prediction models have been evaluated for their potential to accelerate genetic gain for multiple traits, no individual genomic prediction model has outperformed all others across all applications. As an alternative approach, ensembles of multiple individual genomic prediction models can be applied to utilize the complementary strengths of individual prediction models and offset the prediction errors of each. We used the EasiGP (Ensemble AnalySis with Interpretable Genomic Prediction) pipeline to investigate the performance of an ensemble approach, targeting flowering-time traits measured in 2 maize nested association mapping datasets. For both datasets, the ensemble-based prediction approach achieved higher prediction accuracy and lower prediction error across the flowering-time traits compared to each individual model. Multiple genomic regions known to contain key flowering-time-related genes were repeatedly included as features across individual genomic prediction models, indicating the models successfully captured SNPs as features that are associated with genomic regions known to contain flowering-time genes. Although repeatability was high for some genomic regions, estimated marker effects varied across many genomic regions, suggesting that the models might also have captured different aspects of the genetic variation underlying the traits. The ensemble combination of the diverse views likely contributed to the improvement of prediction performance by the ensemble-based approach over the individual prediction models. Ensemble-based prediction can be applied to overcome limitations observed in the continuous exploration for the best individual genomic prediction models that can consistently achieve the highest prediction performance, thereby potentially contributing to improved prediction accuracy for applications in crop breeding.

## Introduction

Crop breeding programs have targeted the development of more resilient genotypes for harsh biotic and abiotic stress conditions (Kholová et al. 2021; Cooper and Messina 2023; Werner et al. 2025). The pressures of climate change have amplified such stresses in agricultural environments, resulting in increasing demand for better-adapted crop genotypes (Ceccarelli et al. 2010; Langridge et al. 2021). Consequently, breeders have endeavored to continuously select genotypes with improved combinations of the target traits to accelerate genetic gain (Messina et al. 2023; Pixley et al. 2023).

Genomic selection (Meuwissen et al. 2001; Bernardo and Yu 2007; Voss-Fels et al. 2019; Khaipho-Burch et al. 2023; Crossa et al. 2025; Escamilla et al. 2025) is a key approach to accelerate genetic gain. The prediction of trait phenotypes using genomic markers can be used to reduce the length of each breeding cycle and therefore the cost of breeding programs (Heffner et al. 2010). Genomic prediction models are trained to estimate the effect of each genomic marker for target trait prediction by capturing patterns of genetic variation associated with phenotypic differences in the reference population of the breeding program (Alemu et al. 2024). Genomic prediction models that can identify repeatable associations between marker combinations and trait variation across populations have the potential to improve the prediction performance for multiple breeding applications (Hammer et al. 2006; Cooper et al. 2014a, 2014b; Voss-Fels et al. 2019; Diepenbrock et al. 2022).

Numerous genomic prediction models have been investigated across a wide range of applications to crop breeding programs (Cooper et al. 2014a, 2014b; Diepenbrock et al. 2022; John et al. 2022; Crossa et al. 2025; Escamilla et al. 2025). However, none of the proposed approaches has shown superior performance for all traits and datasets. For example, Heslot et al. (2012) showed an absence of a consistent best model across 8 prediction models (parametric and machine learning) applied to wheat, barley, and maize datasets. Similarly, Plavšin et al. (2022) evaluated 8 prediction models for quality traits in wheat, revealing that while ridge regression best linear unbiased prediction (rrBLUP) reached the overall highest performance, the differences from other prediction models were small, and the most accurate genomic prediction model was dependent on the environment and the trait. Genomic prediction performance was investigated comprehensively by Washburn et al. (2025); the global genotype-by-environment (G × E) prediction competition evaluated a wide range of genomic prediction models from 128 teams for predicting maize yield traits. Their results showed that no individual genomic prediction model consistently improved prediction performance. Instead, an ensemble of parametric and machine learning models reached the overall highest prediction performance. Other empirical studies (Meher et al. 2022; Lourenço et al. 2024; Gibbs et al. 2025) that also compared the prediction performance of individual genomic prediction models consistently found that no prediction model was superior to others across different prediction scenarios created by combinations of environments, crops, and datasets. These observed results demonstrate the limitations in the development of a single genomic prediction model that is stably generalized with high prediction performance in new environments under cross-validation settings.

The lack of a best individual genomic prediction model could be a consequence of The No Free Lunch Theorem (Wolpert and Macready 1997), which states that the mean performance of prediction models becomes equivalent across all scenarios. The lack of a consistently superior model is likely a consequence of individual prediction models being well-suited for specific scenarios, but not for all. Hence, the continuous exploration for an all-rounder individual genomic prediction model may not be an optimal approach to improve overall prediction performance.

Alternatively, as shown by Washburn et al. (2025), multiple genomic prediction models can be combined as an ensemble to improve prediction performance rather than implementing them independently (Cooper et al. 2025; Messina et al. 2025). For instance, Nascimento et al. (2024) successfully improved genomic prediction performance for several traits, such as yield and total number of fruits, in an Arabica coffee breeding program using ensembles of diverse prediction models. The success of ensembles can be theoretically explained by The Diversity Prediction Theorem (Hong and Page 2004; Page 2007, 2018); the use of diverse prediction algorithms in a Many-Model ensemble lowers the prediction error more than the mean prediction error of individual prediction models. Hence, the prediction error of an ensemble depends on the evaluated prediction errors of individual models included in the ensemble and the diversity of their predictions (Messina et al. 2025; Tomura et al. 2025a).

**Fig. 1.**
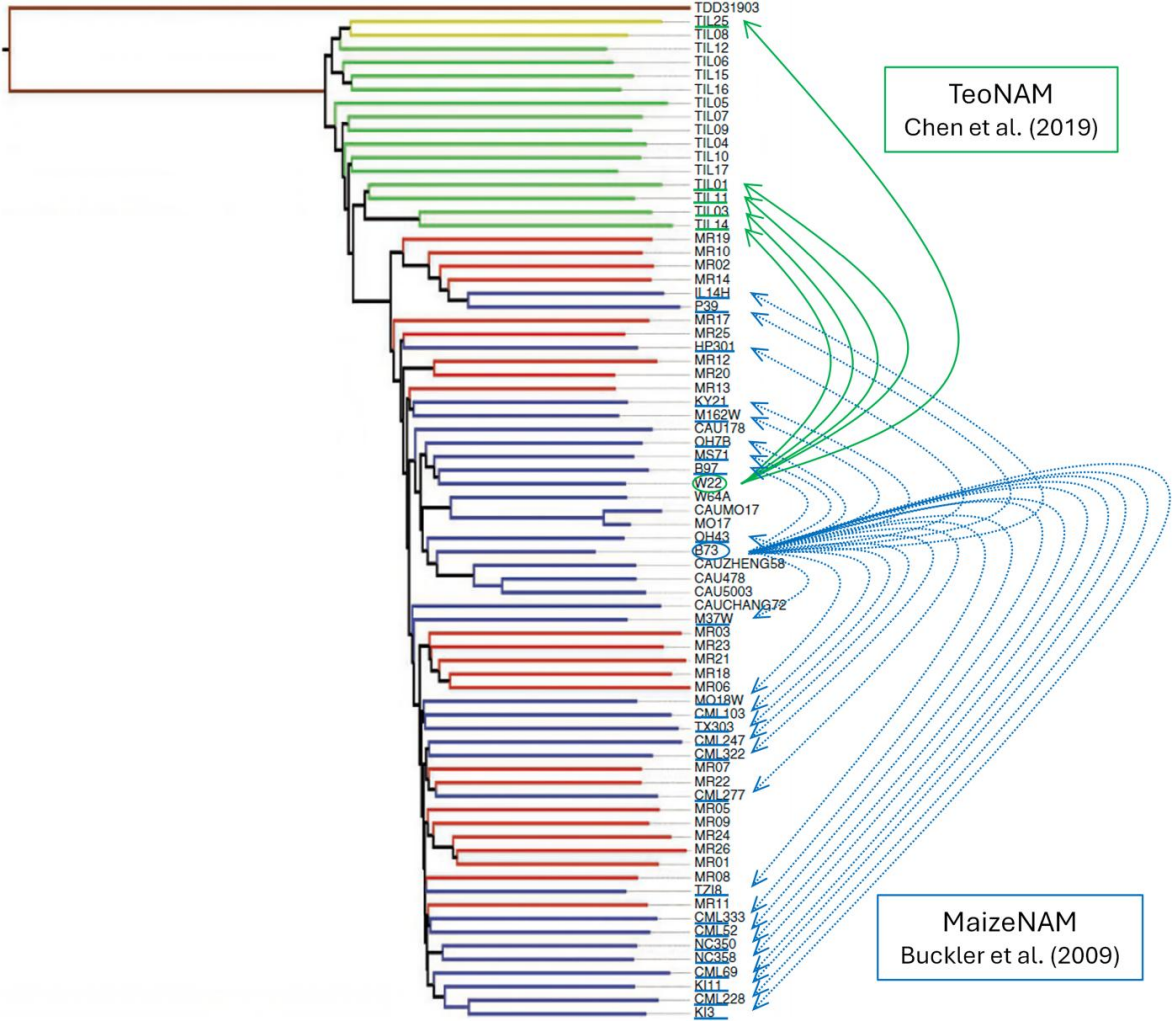
Neighbor-joining tree showing sequence relationships between TeoNAM and MaizeNAM parents [adapted from Hufford et al. (2012) with permission from Professor Jeffrey Ross-Ibarra]. Within the tree, each color represents *parviglumis* (green), landraces (red), improved lines (blue), *mexicana* (yellow), and *Tripsacum* (brown), respectively. Circles around W22 and B73 represent the common parent in the TeoNAM (Chen et al. 2019) and MaizeNAM (Buckler et al. 2009) datasets, respectively. Green and blue lines under several maize line names show donor parents for the TeoNAM and MaizeNAM datasets, respectively. For the TeoNAM dataset, W22 was crossed (green arrows) with 5 donor parents. For the MaizeNAM dataset, B73 was crossed (blue dash arrows) with 25 donor parents.

Using the framework of the Diversity Prediction Theorem, here we investigated whether an ensemble of diverse genomic prediction models improved prediction performance by predicting maize flowering-time traits in 2 well-documented nested association mapping (NAM) examples (Buckler et al. 2009; Chen et al. 2019). The parents of these 2 NAM datasets represent a wide range of maize genetic diversity (Fig. 1), extending from domestication to modern inbred lines used for hybrid breeding (Hufford et al. 2012). This study was guided by 2 objectives. Firstly, the prediction performance of the ensemble was compared with individual genomic prediction models to evaluate the expectations of the Diversity Prediction Theorem for prediction performance improvement. Secondly, the estimates of genomic marker effects on the target traits from each genomic prediction model were compared to observe the association between the diversity in quantified marker effect values and the prediction performance of the ensemble.

## Materials and methods

### Datasets

The TeoNAM dataset (Chen et al. 2019) is a collection of samples from the 5 recombinant inbred line (RIL) populations between the maize line W22 and 5 teosinte types (TIL01, TIL03, TIL11, and TIL14 from *Zea mays* ssp. *parviglumis* and TIL25 from *Z. mays* ssp. *mexicana*). In each RIL population, the F1 was backcrossed with W22. After the backcross was completed, each population was self-pollinated 4 times to develop the RIL populations. A randomized complete block design was applied to test the RILs for each population contributing to the TeoNAM experiment. Each population was tested twice at the University of Wisconsin West Madison Agricultural Research Station. For W22TIL01, W22TIL03, and W22TIL11, the RILs were tested in the summers of 2015 and 2016. For W22TIL14, the RILs were tested in the summers of 2016 and 2017, and for W22TIL25, the RILs were tested in 2 different blocks in the summer of 2017.

The MaizeNAM dataset (Buckler et al. 2009) consists of samples from 25 RIL populations derived from crosses between the maize line B73 and a diverse set of 25 inbred lines from temperate and tropical regions. The 25 RIL populations were developed through self-pollination to the F5 generation. Each population was tested in the following locations during the summer in the United States: Aurora in New York, Clayton in North Carolina, Columbia in Missouri, and Urbana in Illinois. Experiments were conducted twice at each location with a randomized design, and hence, each population was evaluated in the 8 environments. The recorded phenotype scores in each environment were used to estimate the best linear unbiased predictions (BLUPs) with ASReml (v2.0) (Butler et al. 2017) in each population. The best linear unbiased estimates (BLUEs) were calculated by unshrinking the BLUPs per population using heritability, after removing the mean phenotype value for each population (Supplementary Fig. 1). The combination of mean phenotype values and heritability uniquely characterized each population, showing mild positive correlations as the overall trend (Supplementary Fig. 2). In the main manuscript, BLUEs were employed as the target trait phenotypes of each RIL in each prediction scenario for the MaizeNAM dataset. The comparable prediction results and analysis for the BLUPs are reported in Supplementary Material (Supplementary Figs. 3, 10 and 11). The total number of genomic markers (SNPs) and RIL samples from each target dataset is summarized in Table 1 for the dataset level and Supplementary Table 1 for the population level.

**Table 1.** The total number of genomic markers (SNPs) before and after linkage disequilibrium (LD) filtering and the number of recombinant inbred line (RILs) records for the TeoNAM and MaizeNAM datasets.

Dataset	SNPs		RIL records
	Original	LD filtering	
TeoNAM	11,396–16,110	268–342	438–616
MaizeNAM	1,106	170–251	126–196

The selection of the 2 maize NAM datasets was motivated by the genetic diversity that covered a continuum of maize lines from wild relatives to post-domestication lines (Fig. 1; Hufford et al. 2012). The TeoNAM dataset contains the highest level of genetic diversity: Crosses of a temperate maize inbred with representatives of its ancestral species, Teosinte, can uncover components of trait genetic variation fixed during domestication. In contrast, the MaizeNAM dataset is less diverse, as crosses are limited to within a set of domesticated maize lines. Using these 2 maize NAM datasets, characterized by different levels of genetic diversity, we investigated the distinct characteristics of the genomic prediction models for these datasets to understand the predictive behavior of the individual models and their ensemble.

### Data preprocessing

We applied the same data preprocessing methods reported by Tomura et al. (2025a). Briefly, for the TeoNAM dataset, missing SNP markers were imputed by assigning genomic marker values based on flanking markers. RIL samples with missing genomic markers for an entire chromosome and those without phenotype values were removed. For the MaizeNAM dataset, missing SNP markers were imputed with flanking markers and missing phenotypes were removed, as reported by Buckler et al. (2009). Hence, no further imputation was required. Prior to the ensemble-based analyses, the total number of SNPs was reduced further based on linkage disequilibrium (LD) using PLINK (v1.9) (Chang et al. 2015). Any SNP with a squared correlation of 0.8 or above was removed using a window size of 30 kb and a step size of 5 SNPs in both datasets.

The 2 flowering-time-related traits, days to anthesis (DTA) and anthesis to silking interval (ASI), were measured in both datasets. For the TeoNAM dataset, the DTA and ASI traits for each RIL were recorded across 2 environments as reported by Chen et al. (2019). These environments were concatenated for the ensemble genomic prediction analysis following Tomura et al. (2025a), combining genotype and phenotype data from 2 environments into a single dataset with a factor indicating the corresponding environment for each RIL population dataset.

### Genetic variance component calculation

Both additive and non-additive (epistatic) variance components were estimated per population for both DTA and ASI in each dataset following Yadav et al. (2021, 2023). Each variance component was calculated by constructing the respective genomic relationship matrices. The additive genomic relationship matrix ($G_{A}$) was defined below (Yang et al. 2010):


(1) $$\begin{array}{c} G_{A}=\frac{WW^{\prime }}{n} \end{array}$$

where *W* is the incidence matrix corresponding to the additive marker effects and *n* is the number of individuals. The epistatic genomic relationship matrix ($G_{AA}$) was developed by the Hadamard product (⨀) of the additive genomic relationship matrix as defined below:


(2) $$\begin{array}{c} G_{AA}=\frac{G_{A}⊙G_{A}}{\frac{tr(G_{A}⊙G_{A})}{n}} \end{array}$$

where tr(⋅) is the trace that sums the diagonal elements in the matrix. Using the $G_{A}$ and $G_{AA}$ matrices, extended genomic best linear unbiased prediction (extended-GBLUP) was modeled:


(3) $$\begin{array}{c} y^{n*1}=X\beta +Wa+Wt+\varepsilon \end{array}$$

where *y* is the vector of predicted phenotypes, *X* is the incidence matrix assigned to fixed effects *β*, *W* is the incidence matrix for the random effects, $a∼N(0,G_{A},\sigma_{A}^{2})$ is the vector of additive genomic marker effects, $t∼N(0,G_{AA},\sigma_{E}^{2})$ is the vector of epistatic genomic marker effects, and $\varepsilon ∼N(0,I,\sigma_{\varepsilon }^{2})$ is the vector of random residual effects. The variance of the additive genomic marker effects ($\sigma_{A}^{2}$), epistatic genomic marker effects ($\sigma_{E}^{2}$), and random residual effects ($\sigma_{\varepsilon }^{2}$) was summed for the total variance. Each variance component was then expressed as a proportion of the total variance, yielding values between 0 and 1. For the TeoNAM dataset, variance components were calculated per environment and averaged within each population. For the MaizeNAM dataset, variance components were directly calculated at the population level for average performance across environments, since data for each RIL population were not available for each environment. The extended-GBLUP was implemented by ASReml-R (v4.2).

### Genomic prediction model implementation and genomic level analysis

Six individual genomic prediction models were implemented and their inferred marker effects for prediction were extracted using the computational pipeline tool, EasiGP (Tomura et al. 2025b). Following Tomura et al (2025a), 3 conventional genomic prediction models [rrBLUP (Meuwissen et al. 2001), BayesB (Meuwissen et al. 2001), and reproducing kernel Hilbert space regression (RKHS; Gianola and Van Kaam 2008)] and 3 machine learning models [random forest (RF; Breiman 2001), support vector regression (SVR; Drucker et al. 1996) and multi-layer perceptron (MLP; Rosenblatt 1958)] were applied in this study. For rrBLUP, the genomic marker effects are assumed to be normally distributed for all genomic markers. In contrast, some genomic marker effects can be shrunk to zero rather than forming a normal distribution in BayesB (Endelman 2011). BayesB has been widely applied to genomic prediction for crop breeding and hence used as a benchmark model among the Bayesian alphabet models in this study. For RKHS, genomic markers are mapped to the Hilbert space using a kernel that estimates the mean distance between genotypes as squared Euclidean distance (del los Campos et al. 2009). RF consists of numerous decision trees developed from a fraction of the given dataset. Each decision tree returns prediction values by traversing the tree with input attribute values. The mean predicted value from all the decision trees was calculated as the final prediction value. SVR predicts values by optimizing the position of a hyperplane that maximizes the number of samples included in the space defined as an epsilon tube. The size of the epsilon tube is determined by the space between the hyperplane and the location of support vectors that create the outer boundary of the tube as the decision boundary (Drucker et al. 1996). Hence, the hyperplane is optimized in consideration of adjusting the position of support vectors. MLP mimics human brain systems by continuous nonlinear aggregation of input data from neighboring neurons in a forward direction (Dave and Dutta 2014). Those 6 prediction models have been widely used for genomic prediction in crop breeding and the 3 prediction models from each category, conventional (including parametric and semi-parametric) and machine learning, were chosen for balanced comparison. The ensemble-average model calculates the mean predicted phenotypes from the predictions returned by the 6 individual models with equal weights. The genomic prediction models constructed in this study were trained using genomic markers in a numerical format to output predicted phenotypes for each prediction scenario. The details of the prediction scenarios are discussed in “Model evaluation”.

The following hyperparameter values were assigned to each individual genomic prediction model. For rrBLUP, BayesB, and RKHS, the number of iterations was set as 12,000 and the burn-in was 2,000 for the 3 models in both datasets. Default values were used for all other parameters. For RF and SVR, the default hyperparameter values were employed for both prediction models in both datasets, except for the number of trees (1,000) in RF. MLP was structured with 1 hidden layer in both datasets. For the TeoNAM dataset, 50 neurons were included in the hidden layer with a dropout of 0 and activated by the Rectified Linear Unit (ReLU; Nair and Hinton 2010) function. The model was trained for 200 epochs with a learning rate of 0.005 using the Adaptive Moment Estimation with Weight Decay (AdamW; Loshchilov and Hutter 2017) optimizer. For the MaizeNAM dataset, the number of neurons was reduced to 10 with a dropout of 0.1 and also activated by the ReLU function. The model was trained for 2,500 epochs with a learning rate of 0.005 using Root Mean Square Propagation (RMSprop; Tieleman 2012). The selected hyperparameter values reached the highest prediction performance among the combinations in each dataset from our heuristic investigation of optimum hyperparameter value combinations. The detailed associations between the selected hyperparameter values of the individual genomic prediction models and the prediction performance of ensembles can be further investigated as a future research area.

The effect of each marker on trait phenotype prediction was estimated after the development of genomic prediction models as a part of the pipeline in EasiGP. For rrBLUP and BayesB, the allele substitution effects were inferred as marker effects. For RF, marker effects were inferred using feature importance based on the impurity-based approach (Ishwaran 2015). The importance of a feature is determined by the total impurity value derived from the impurity value of each decision node for the feature. Higher importance is allocated to a feature that can clearly divide the data points (RILs) into sub-decision nodes, clustering data points with similar labels (observed phenotypes). For RKHS, SVR, and MLP, Shapley scores (Shapley 1953; Lundberg and Lee 2017) were used as inferred marker effects. Shapley scores in this study estimate the conditional effect of a particular genomic marker. Since Shapley scores are calculated cumulatively by considering possible genomic marker combinations, Shapley scores were also applied to RF at the pairwise level to infer genomic marker-by-marker interaction effects. For the ensemble-average model, the mean values of the normalized marker effects from all the individual genomic prediction models were calculated with the same weights.

The inferred marker effects from each genomic prediction model and the ensemble were mapped to corresponding marker regions using circos plots (Krzywinski et al. 2009) in the final phase of EasiGP. The generated circos plots also visualized the genome positions of known genes and regulators of DTA and QTL reported from 3 previous studies: Chen et al. (2019) for the TeoNAM dataset and Buckler et al. (2009) for the MaizeNAM dataset. Wisser et al. (2019) identified several key genes affecting flowering time in maize from an empirical selection experiment investigating the short-term evolution of tropical region maize lines. Lastly, Dong et al. (2012) created a list of key gene regions and their interactions for flowering time in maize. They classified key gene regions into 2 categories [leaf and shoot apical meristem (SAM)] based on the tissue or organ of the maize plant where they act to determine flowering time. These genes were also classified into 7 categories based on their a priori defined functions (light transduction, clock, photoperiod, autonomous, aging, gibberellin, and integrator).

### The Diversity Prediction Theorem

The Diversity Prediction Theorem compares the error of the ensemble-average model against the mean error of individual prediction models in relation to the diversity of the predicted values (Hong and Page 2004; Page 2007, 2018):


(4) $$\begin{array}{c} {\left(\bar{M}-V\right)}^{2}=\overset{N}{\underset{i=1}{\sum }}\frac{{\left(M_{i}-V\right)}^{2}}{N}-\overset{N}{\underset{i=1}{\sum }}\frac{{\left(M_{i}-\bar{M}\right)}^{2}}{N} \end{array}$$

where $M_{i}$ is the predicted value from the prediction model *i*, $\bar{M}$ is the mean predicted values from the *i* individual prediction models, *V* is the true value, and *N* is the total number of prediction models considered. The Many-Model error (1st term) is calculated by subtracting the prediction diversity (3rd term) from the mean error (2nd term). In our study, an observed phenotype was defined as the true value *V* and the Many-Model error was regarded as the ensemble error. $\bar{M}$ was calculated by averaging the predicted phenotypes from the 6 individual genomic prediction models used in this study.

Since the scale of phenotypes varied across the traits and datasets, each term value in the Diversity Prediction Theorem was not directly comparable. Hence, the coefficient of variation (CV) was estimated in each term to be used as a metric to measure the diversity level of predicted phenotypes for the ensemble in each prediction scenario:


(5) $$\begin{array}{c} {\hat{CV}}_{term_{j}}=\;\frac{\sigma_{term_{j}}}{\mu_{term_{j}}} \end{array}$$

where ${\hat{CV}}_{term_{j}}$ is an estimate of CV for term *j* in the Diversity Prediction Theorem, the $\sigma_{term_{j}}$ represents the standard deviation of the term *j* in the theorem, and $\mu_{term_{j}}$ represents the mean value of the term *j* in the theorem. A larger estimated CV for the 3rd term of Equation (4) indicates higher diversity in the predicted phenotypes and hence individual genomic prediction models included are considered to be more diverse. The CV was estimated per trait for each dataset.

### Model evaluation

The individual and ensemble genomic prediction models were evaluated by iterative prediction scenarios with different settings. For the TeoNAM dataset, 3 different training-test set ratios (0.8–0.2, 0.65–0.35, and 0.5–0.5) were applied to mitigate the data size effect. In each population-training-test-set scenario, RIL data were randomly sampled 500 times. Hence, 7,500 prediction scenarios (5 populations * 3 ratios * 500 samples) were developed per trait. The same 3 training-test set ratios were used in the MaizeNAM dataset. For each of the 25 populations in the dataset, the RILs were sampled 50 times for each population-training-test-set scenario, resulting in 3,750 prediction scenarios (25 populations * 3 ratios * 50 samples) per trait. In each prediction scenario, the genomic prediction models were trained, and their prediction performance was evaluated using the Pearson correlation coefficient and mean squared error (MSE). After recording the prediction performance metrics, the developed genomic prediction models were discarded, treating each prediction scenario as an independent prediction task. Using the recorded predicted phenotypes from the genomic prediction models, the 3 components of the Diversity Prediction Theorem [Equation (4)] were calculated for the ensemble. At the end of the iterative predictions process, the median values were calculated for both the Pearson correlation coefficients and MSE to develop violin plots, whereas the mean and CV values were calculated for the 3 terms of the Diversity Prediction Theorem.

## Results

### Maize NAM datasets and traits showed a distinctive composition of genetic variance components

**Fig. 2.**
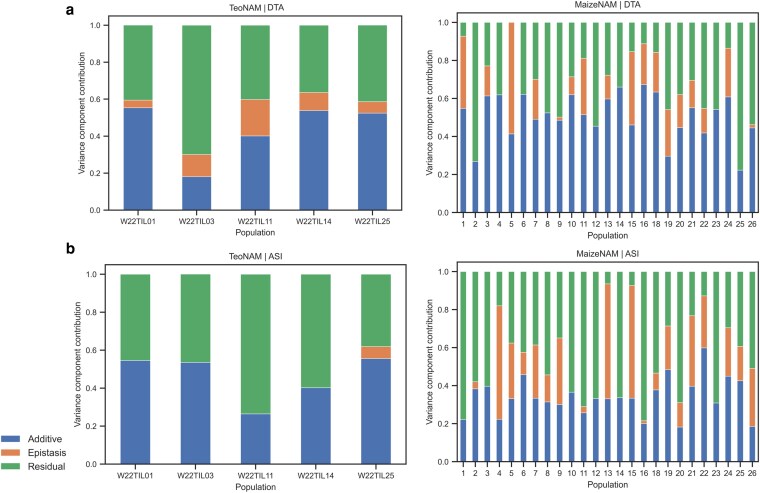
The variance components of each population in the TeoNAM and MaizeNAM datasets for the a) days to anthesis (DTA) and b) anthesis to silking interval (ASI) trait. The relative magnitudes of variance components partitioned into additive (blue), epistasis (orange), and residual (green) effects.

The estimated genetic variance components for the traits provide a preliminary characterization of the 2 target datasets (Fig. 2). For ASI, the median genetic variance component (additive and epistasis) value for the TeoNAM dataset (0.535) was higher than for the MaizeNAM dataset (0.490), indicating that a larger proportion of the phenotypic variance for ASI was explained by genetic effects in the TeoNAM dataset. The median value was used since some populations showed extreme variance component values. This observation is aligned with the genetic diversity previously estimated in the parent lines in the TeoNAM and MaizeNAM datasets; nucleotide diversity (*π*) across non-overlapping 10-kb windows was higher for the Teosinte lines used in the TeoNAM populations (*π* = 0.0059; *Z. mays ssp. parviglumis*), compared to the improved maize lines included as parental lines in several MaizeNAM populations (*π* = 0.0048) (Hufford et al. 2012). In contrast, there was a higher effect of the median genetic variance component for DTA in the MaizeNAM dataset (0.659) compared to the TeoNAM dataset (0.594), indicating that genetic effects explained a larger proportion of the phenotypic variance in the MaizeNAM dataset for DTA. For both datasets, the sum of both additive and epistasis variance components for ASI was smaller than for DTA. This observation likely corresponds to the high environmental plasticity of ASI captured in the residual component (Buckler et al. 2009; Silva et al. 2022). Such a difference in the variance components indicates the potential for distinctive predictive behaviors of the genomic prediction models for the prediction scenarios characterized by traits and datasets, investigated in the following subsections.

### Ensemble prediction showed improved prediction performance in comparison to the individual genomic prediction models

**Fig. 3.**
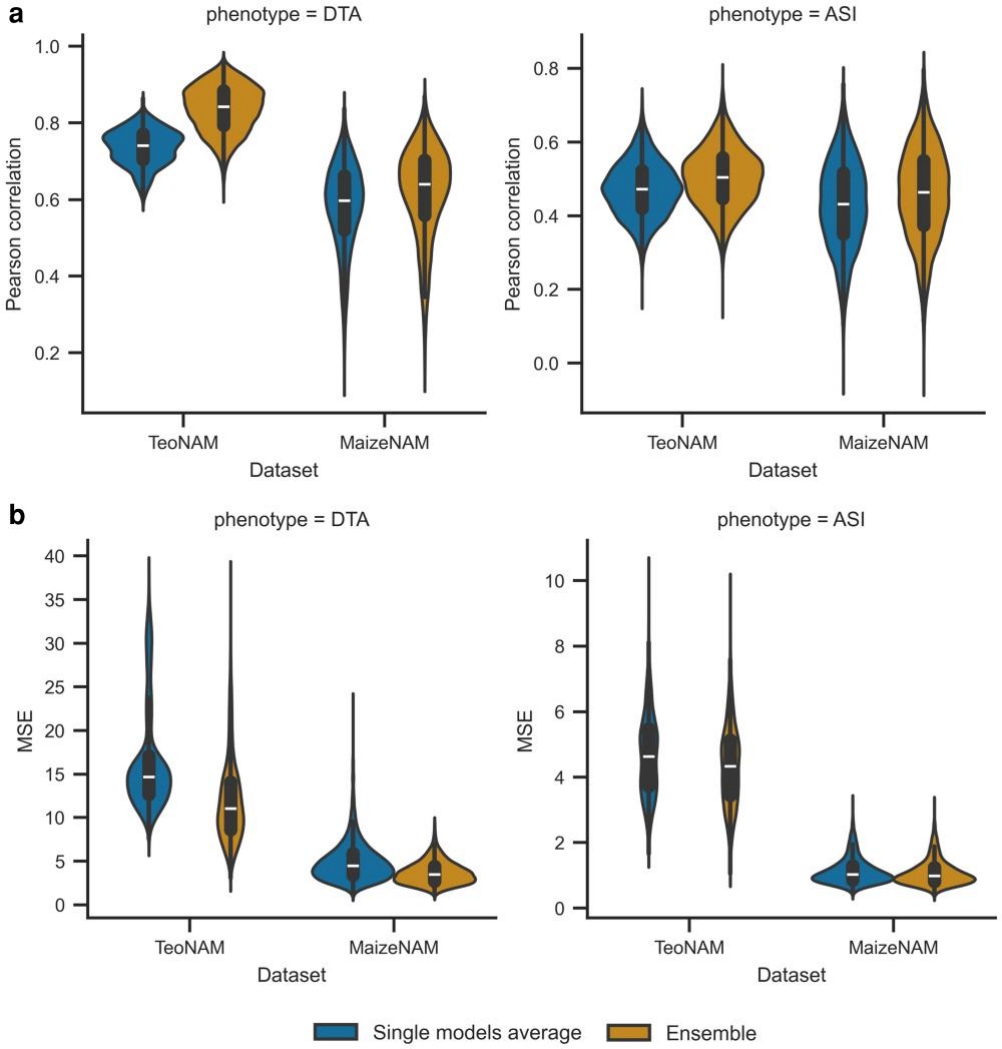
The comparison of prediction performance between the average performance of the individual genomic prediction models (single models average) and the ensemble-average model (ensemble) for the days to anthesis (DTA) and anthesis to silking interval (ASI) traits in the TeoNAM and MaizeNAM datasets. The prediction performance was measured using the 2 metrics: a) Pearson correlation and b) mean squared error (MSE). The prediction performance was measured in 7,500 prediction scenarios for the TeoNAM dataset and 3,750 prediction scenarios for the MaizeNAM dataset for each trait. The prediction scenarios were generated by the combination of the 3 training-test ratios (0.8–0.2, 0.65–0.35, and 0.5–0.5), populations and sampling numbers. The width of the violins represents the distribution of performance metrics. The white horizontal lines on the black box plots show the median value for each metric. The whiskers extend 1.5 times the interquartile range.

The ensemble-average model improved the prediction performance across the target traits and datasets relative to the mean prediction performance of individual genomic prediction models by increasing prediction accuracy and reducing prediction error (Fig. 3). For the TeoNAM dataset, the ensemble-average model reached higher median prediction accuracy for DTA (0.842) and ASI (0.505) in contrast to the mean prediction accuracy of the individual genomic prediction models for DTA (0.741) and ASI (0.473). The ensemble-average model also had a smaller median prediction error for DTA (the ensemble-average model = 11.033 and the mean of individual genomic prediction models = 14.699) and ASI (the ensemble-average model = 4.339 and the mean of individual genomic prediction models = 4.638). For the MaizeNAM dataset, the median prediction accuracy for DTA and ASI was higher for the ensemble-average model (0.640 and 0.464, respectively) compared to the mean performance of the individual genomic prediction models (0.598 and 0.432, respectively). Similarly, there was improvement in the median prediction error for both traits (DTA = 3.471 and ASI = 0.980 in contrast to DTA = 4.467 and ASI = 1.023). This consistent outperformance of the ensemble-average model was also observed in the predictions using BLUPs for the MaizeNAM dataset, showing a similar median prediction performance for the BLUEs in both traits (Supplementary Fig. 3). The observed results indicated that the ensemble-average model consistently outperformed the mean prediction performance of the individual models.

Higher prediction performance of the ensemble-average model was also observed in the comparison with the individual genomic prediction models (Supplementary Fig. 4), achieving the highest prediction performance or equivalent performance to the best individual genomic prediction models throughout the trait-by-dataset prediction scenarios in this study. The ensemble-average model was selected as one of the top 3 best genomic prediction models, competing with rrBLUP, BayesB, and RKHS, in the range of generated prediction scenarios from this study. When the prediction performance of the genomic prediction models was analyzed at the population level in each dataset (Supplementary Table 2), the ensemble also showed stable improved prediction performance by being included as one of the top 3 prediction models in trait-by-population prediction scenarios. For prediction accuracy, the ensemble showed the highest percentage (78.3%) of achieving the top 3 most accurate prediction models measured in terms of Pearson correlation, followed by BayesB (75%) and rrBLUP (73.3%). For the prediction error, rrBLUP and BayesB reached the highest percentage of achieving the top 3 lowest median MSE (78.3%), followed by RKHS (68.3%) and ensemble (65.0%). In contrast, the machine learning models (RF, SVR, and MLP) remained at lower prediction performance throughout the trait-by-population prediction scenarios in both prediction accuracy and error by consistently having a low percentage (less than 10.0%). The stability in the improved prediction performance of the ensemble was also observed in trait-by-training set ratio prediction scenarios in each dataset (Supplementary Fig. 5), showing constant performance reduction rate in proportion to the reduction in training set size along with rrBLUP, BayesB, RF, and SVR. The ensemble minimized the effect of the rapid performance reduction that was observed for RKHS and MLP, attributed to the training set size reduction in some prediction scenarios. The comparison with the individual genomic prediction models demonstrated that the ensemble-average model consistently reached a higher prediction accuracy across a wide range of prediction scenarios.

### Diversity contributed to the performance improvement of the ensemble

The level of prediction improvement of the ensemble varied with traits and datasets, associated with the level of diversity in predicted phenotypes. The CV for the 3rd term was higher in the TeoNAM dataset for both traits (DTA = 1.99 and ASI = 2.15) in contrast to the MaizeNAM dataset (DTA = 1.80 and ASI = 1.15) (Table 2). A higher CV for the 3rd term indicates that the predicted phenotypes from the individual genomic prediction models are more diverse. Since greater improvements in prediction performance were observed in the TeoNAM dataset compared to the MaizeNAM dataset, the higher CV values for both DTA and ASI in the TeoNAM dataset were associated with higher diversity in the predicted phenotypes from the individual genomic prediction models. This finding indicated that there was a positive association between the level of diversity in the predicted phenotypes and the prediction performance of the ensemble.

**Table 2.** Measurement of diversity in individual genomic prediction models included in the ensemble models using the framework of the Diversity Prediction Theorem. The mean value of each term in the Diversity Prediction Theorem was calculated for the days to anthesis (DTA) and anthesis to silking interval (ASI) traits in the TeoNAM and MaizeNAM datasets. The ensemble (Many-Model) error, mean error, and prediction diversity represent the 1st, 2nd, and 3rd terms in the theorem, respectively. In each dataset, “Mean” represents the actual mean term value, whereas “CV” indicates the coefficient of variation for each term value. The sign “±” indicates the standard error of each corresponding mean value.

Traits	Terms	TeoNAM		MaizeNAM	
		Mean	CV	Mean	CV
DTA	Ensemble error	12.77 ± 0.021	1.90	3.70 ± 0.013	1.73
	(1st term)				
	Mean error	37.62 ± 0.054	1.66	5.07 ± 0.014	1.38
	(2nd term)				
	Prediction diversity	24.85 ± 0.043	1.99	1.37 ± 0.005	1.80
	(3rd term)				
ASI	Ensemble error	4.43 ± 0.010	2.44	1.08 ± 0.004	1.69
	(1st term)				
	Mean error	5.03 ± 0.010	2.19	1.13 ± 0.004	1.62
	(2nd term)				
	Prediction diversity	0.60 ± 0.001	2.15	0.05 ± 0.000	1.15
	(3rd term)				

**Fig. 4.**
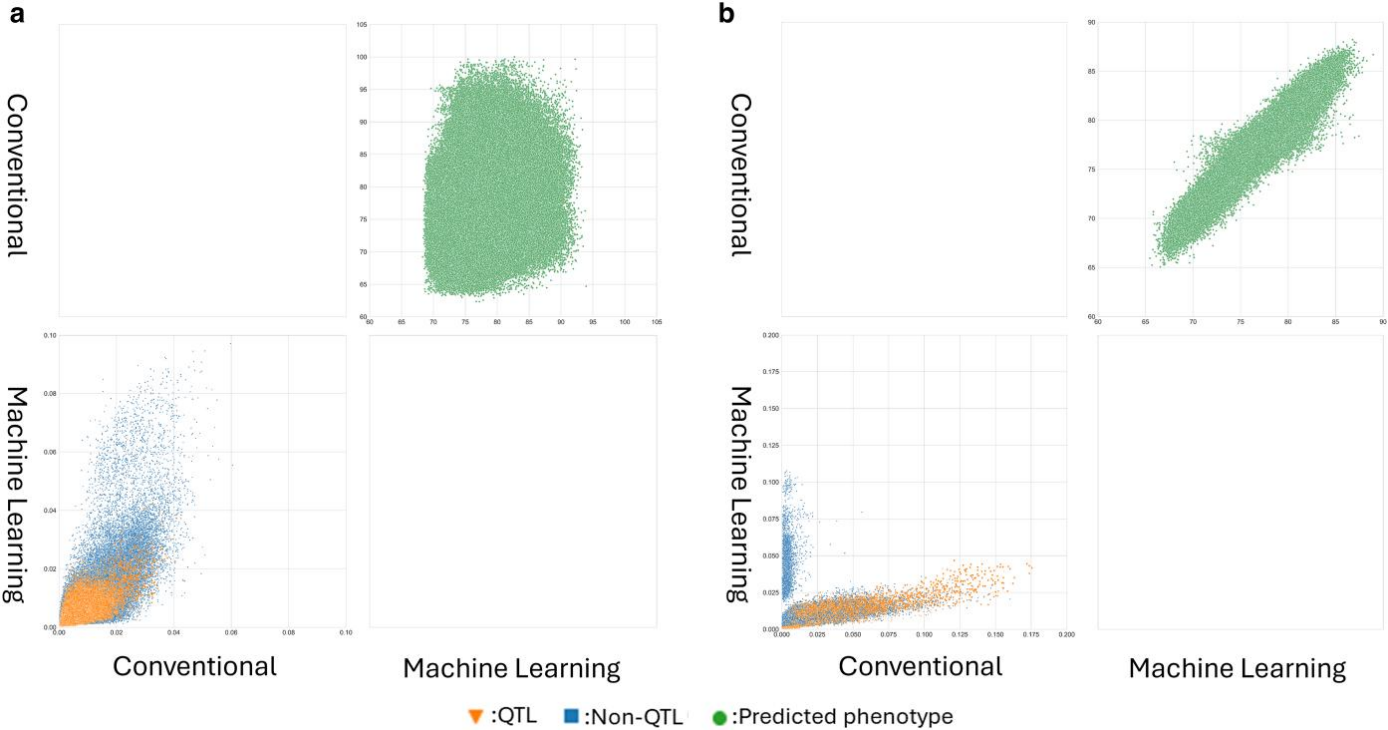
Pairwise comparisons of the conventional (rrBLUP, BayesB, and RKHS) and machine learning models (RF, SVR, and MLP) for the days to anthesis (DTA) trait across all the prediction scenarios for the a) TeoNAM (7,500 prediction scenarios) and b) MaizeNAM (3,750 prediction scenarios) datasets. The prediction scenarios were generated by the combination of the 3 training-test ratios (0.8–0.2, 0.65–0.35, and 0.5–0.5), populations, and sampling numbers. The genomic prediction model groups were compared for mean predicted phenotypes (top right triangle) and mean normalized genomic marker effects (the bottom left triangle), calculated within each prediction model category. The green dots represent a pair of predicted phenotypes of RIL samples in the test sets for each prediction scenario. The blue squares and orange triangles represent a pair of extracted genomic marker effects from each genomic marker in each sample scenario that were identified as non-QTL and QTL markers, respectively, by Chen et al. (2019) for the TeoNAM dataset and Buckler et al. (2009) for the MaizeNAM dataset.

The comparison of the predicted DTA at both the phenotype and genome marker levels indicated a different degree of information diversity between the datasets and between prediction model groups, conventional (rrBLUP, BayesB, and RKHS) vs machine learning (RF, SVR, and MLP) (Fig. 4). Pairwise comparisons between the conventional models and machine learning models in the MaizeNAM dataset demonstrated stronger positive associations when measured in Pearson correlation (mean predicted phenotypes: *r* = 0.961 and mean inferred marker effects: *r* = 0.431) compared to the TeoNAM dataset (mean predicted phenotypes: *r* = 0.165 and mean inferred marker effects: *r* = 0.603). These results indicate that, for the TeoNAM dataset, the individual genomic prediction models returned more diverse predicted phenotypes, consistent with the substantial variation in marker effect estimates between the prediction model groups. The relatively high diversity in the TeoNAM dataset was also observed for ASI at the phenotype levels (Supplementary Fig. 6). The MaizeNAM showed a higher positive association at the phenotypic level (mean predicted phenotypes: *r* = 0.954 and mean inferred marker effects: *r* = 0.461) compared to the TeoNAM dataset (mean predicted phenotypes: *r* = 0.197 and mean inferred marker effects: *r* = 0.750). This general trend of strong positive associations at the phenotype and genome levels in both traits for the MaizeNAM dataset was also illustrated in the pairwise comparison results for the 6 individual prediction models in both datasets (Supplementary Figs. 7 and 8; Supplementary Tables 3 and 4). The only exception was observed in the pairwise comparison with RKHS at the genome level for the MaizeNAM dataset, which warrants further investigation into the underlying cause in future studies. This higher diversity among the prediction models at both phenotype and genome levels for the TeoNAM dataset likely contributed to the higher level of improvement in prediction performance of the ensemble model in comparison to the MaizeNAM dataset (Figs. 3 and 4).

There were some important differences observed between the conventional and machine learning prediction models at the level of the estimated marker effects. The effects for a significant fraction of the markers were reduced to 0 for the conventional prediction models due to the shrinkage factors incorporated in their prediction mechanisms, while the assigned effects for these markers from the machine learning models did not show such a shrinkage. Consequently, the genomic marker effects were not highly correlated between the conventional and machine learning model groups. This trend was especially emphasized for the MaizeNAM dataset. The comparison of the marker effects between the 2 prediction model groups clearly indicated that many genomic marker effects were captured differently as features between the 2 prediction model groups based on their unique algorithms.

**Fig. 5.**
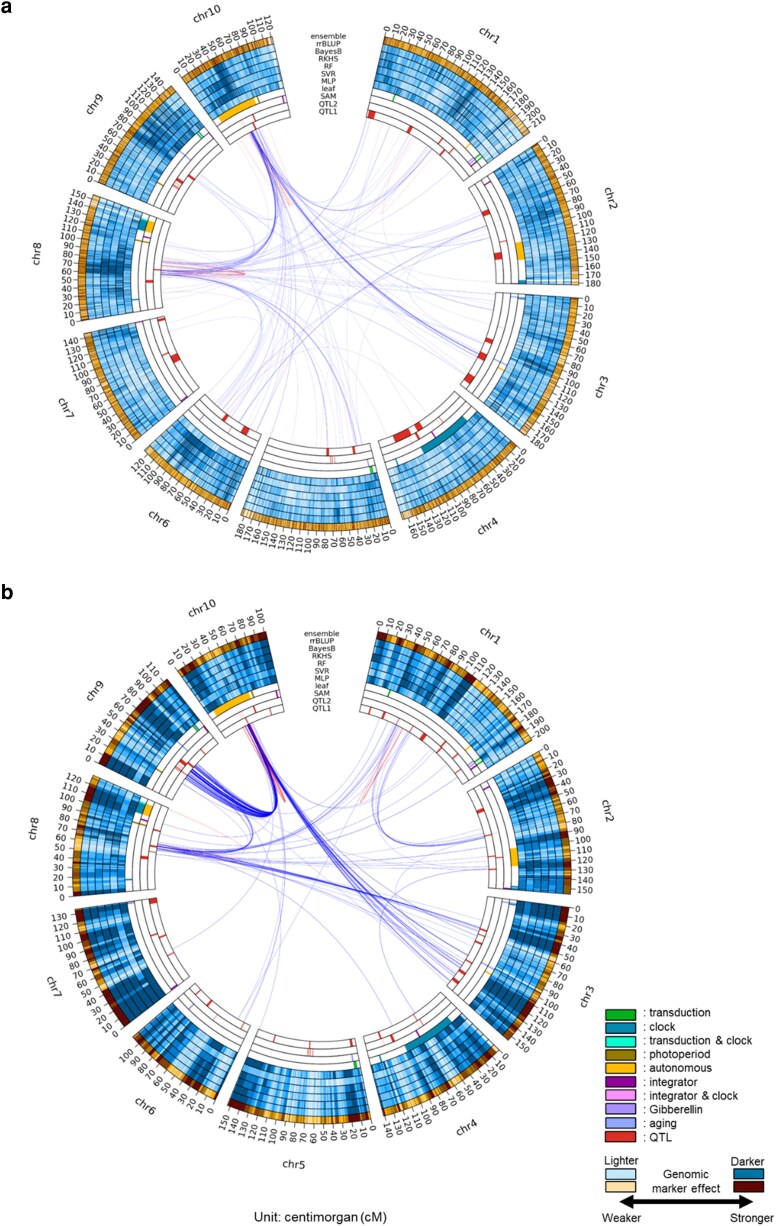
Circos plots for the days to anthesis (DTA) trait using the a) TeoNAM and b) MaizeNAM datasets. The innermost (QTL 1) ring shows the QTL gene regions estimated by Chen et al. (2019) for the TeoNAM dataset and by Buckler et al. (2009) for the MaizeNAM dataset. The 2nd innermost ring (QTL 2) represents the QTL gene regions identified by Wisser et al. (2019). The 3rd and 4th innermost rings represent gene regulators that affect the shoot apical meristem (SAM) and leaf, respectively, identified by Dong et al. (2012). The blue rings (5th to 10th) represent the genomic marker effects across the gene regions estimated by MLP, SVR, RF, RKHS, BayesB and rrBLUP, respectively. The outermost orange ring is the genomic marker effects for the ensemble-average model (ensemble). The numbers at the outermost ring represent genetic distance in centimorgans (cM). The darkness level of the blue and orange colors indicates the strength of the genomic marker effects, sectioned into 10 levels using the quantiles. Darker colors represent higher genomic marker effect levels. The red and blue lines between genome regions are the genomic marker interaction effects calculated by pairwise Shapley scores from RF (top 0.01%; red = within chromosome and blue = between chromosomes).

The diversity of the prediction models at the genome level was further investigated by mapping estimated marker effects from each to corresponding genome regions for both traits (Fig. 5, Supplementary Fig. S9). The highlighted genomic markers and predictive patterns from each genomic prediction model were more variable in the TeoNAM dataset (Fig. 5a, Supplementary Fig. 9a) in comparison to the MaizeNAM dataset (Fig. 5a, Supplementary Fig. 9b), consistent with the expectations of the Diversity Prediction Theorem framework (Table 2) and the results of the ensemble model prediction (Fig. 3). While the different genomic prediction models identified many similar regions of the genome, the effect of each marker identified in these regions varied considerably in the TeoNAM dataset (Fig. 5a, Supplementary Fig. 9a). In the MaizeNAM dataset, on the other hand, there was more consistency across the genomic prediction models (Fig. 5b, Supplementary Fig. 9b). The genomic prediction models detected similar genomic regions with a stronger consensus on the effect of each marker. This observation also highlights the positive association between the prediction performance of the ensembles and the diversity of individual prediction models included in the ensembles at the genome level.

It is also noted that the individual genomic prediction models combine genomic marker effects from both QTL and non-QTL regions to predict target trait phenotypes rather than only using the estimated marker effects from QTL regions identified in the previous analyses of both datasets (Buckler et al. 2009; Chen et al. 2019). For the TeoNAM dataset, while the markers at the peaks of the QTL identified by Chen et al. (2019) were included in the genomic prediction models, strong weights were less frequently assigned by the genomic prediction models (Fig. 4a, Supplementary Fig. S6a, S7a, and S8a; lower triangle of pairwise comparisons). For the MaizeNAM dataset, in contrast, while the genomic prediction models allocated stronger weights to the markers at the peaks of the QTL identified by Buckler et al. (2009) from their analysis of the same dataset, strong marker effects were also assigned to several markers corresponding to non-QTL regions (Fig. 4b, Supplementary Figs. S6b, S7b, and S8b; lower triangle of pairwise comparisons). The individual genomic prediction models not only utilized the association between QTL regions and corresponding traits to predict phenotypes but also included non-QTL regions as features by assigning stronger effect weights to them.

### Several genome regions containing key gene regulators were highlighted by EasiGP

The constructed circos plots showed several genome regions with overlap between the genomic marker regions highlighted by the genomic prediction models and known key gene regulators for the traits (Fig. 5). For DTA, the features of the genomic prediction models were associated with genome regions containing several key maize flowering-time genes in both datasets. For example, in the TeoNAM dataset, the region between 35 cM and 50 cM in chromosome 10 was repeatedly targeted across genomic prediction models (with MLP as an exception). This region contains *ZmCCT10*, involved in regulating flowering time in maize, known to upregulate the circadian clock as part of the photoperiod pathway (Dong et al. 2012; Chen et al. 2019; Wisser et al. 2019). The region containing *ZmCCT10* also showed strong interactions with the region in chromosome 8 (between 60 cM and 80 cM). This region includes *ZCN8*, a well-established gene controlling flowering time in maize that is known to interact with *ZmCCT10* (Dong et al. 2012; Chen et al. 2019; Wisser et al. 2019). In the MaizeNAM dataset, in addition to the same 2 regions identified in the TeoNAM dataset, the genomic prediction models identified another region between 50 cM and 60 cM in chromosome 9 that strongly interacted with the region containing *ZmCCT10* in chromosome 10 and the region containing *ZCN8* in chromosome 8. This chromosome 9 region contains *ZmCCT9* (Wisser et al. 2019), another gene involved in the photoperiod pathway that negatively regulates *ZCN8* (Huang et al. 2018). For ASI, several examples of overlap between the regions highlighted by the genomic prediction models and previously identified QTL regions (Buckler et al. 2009; Chen et al. 2019) were also observed in both datasets (Supplementary Fig. 9). Such examples of overlap between the regions of the genome identified across genomic prediction models and the key gene regulators from the literature in both datasets indicated that these genomic prediction models repeatedly identified regions containing several known causative loci. The stronger consensus in the highlighted feature of marker effects was also observed in the results based on the analyses conducted on BLUPs in the MaizeNAM dataset, with minor differences in the extracted genomic marker-by-marker interactions (Supplementary Figs. 10 and 11). Combined with the observed results from the previous subsection, genomic-level analysis of the prediction models revealed that they repeatedly identified several regions of the genome containing key genes for maize flowering-time traits, while the diverse effects of markers across other genome regions contributed to the predictive performance of the ensembles.

## Discussion

Breeders have continuously searched for prediction methods that can consistently and accurately distinguish individuals with the desired traits in their breeding programs (Cooper et al. 2014a, 2014b; Voss-Fels et al. 2019; Diepenbrock et al. 2022; Crossa et al. 2025; Escamilla et al. 2025). The results from previous genomic prediction research indicate that genomic prediction models can accurately identify superior individuals under certain prediction scenarios (Azodi et al. 2019; Diepenbrock et al. 2022). However, a priori, it is often not possible to obtain information that determines the optimal combination of scenarios and genomic prediction models without a significant trial-and-error process, since no trends or patterns in the combinations are often observed (Jeon et al. 2023; Crossa et al. 2025). For instance, RKHS reached the highest prediction accuracy on wheat yield prediction for 1 wheat dataset (Heslot et al. 2012). However, this trend may not always be observed. In our study, rrBLUP and BayesB showed relatively high prediction performance across different prediction scenarios. The prediction performance superiority for RKHS was not consistent, showing diminished RKHS prediction accuracy at one of the lowest levels for wheat yield traits in another dataset (Heslot et al. 2012). This trend of inconsistency in the best genomic prediction model was observed in other studies as well (Endelman 2011; Meher et al. 2022; Lourenço et al. 2024; Montesinos-López et al. 2024). The overwhelming empirical evidence strongly supports the conclusion of no consistently outperforming individual genomic prediction models.

Machine learning models also failed to consistently improve the prediction performance despite their ability to detect nonlinear prediction patterns, as observed in this study. Heslot et al. (2012) also emphasized lower prediction performance of machine learning models, including RF, SVR, and MLP, compared to conventional genomic prediction models such as rrBLUP and Bayesian approaches for several yield traits in wheat and maize. Their results aligned with the observed results in our study, showing poor prediction performance of RF, SVR, and MLP when used individually. Machine learning approaches did not necessarily outperform the conventional genomic prediction models despite the expectation of outperformance via capturing complex nonlinear prediction patterns incorporated in datasets (Howard and Lipka 2025). A smaller total number of data points (RIL records) in relation to the total number of included genomic markers might have hindered the detection of key prediction patterns, often described as the curse of dimensionality (Bellman 1957). However, MLP reached higher prediction performance than GBLUP in other prediction datasets and traits, including yield traits, in wheat, maize, and rice (Montesinos-López et al. 2025). The current machine learning models can outperform the conventional genomic prediction models for some prediction scenarios, drawing the same conclusion that the most preferable genomic prediction model is highly scenario dependent.

In contrast, the ensemble of multiple individual genomic prediction models consistently improved prediction performance in accordance with previous investigations (Kick and Washburn 2023; Washburn et al. 2025) and the expectations of the Diversity Prediction Theorem, given that the individual genomic prediction models included in the ensembles were diverse (Hong and Page 2004; Page 2018; Messina et al. 2025). Hence, the improvement in prediction performance achieved by the ensemble was marginal in some cases, depending on the diversity level in the predictive information included in the individual prediction models. However, the performance of the ensemble approach was always at least comparable to the mean prediction performance of individual genomic prediction models. The significance of our study arises from connecting the theorem to the empirical investigation, showing that the effect of the theorem is applicable in actual genomic prediction settings. The ensemble of predicted phenotypes through the mean value calculation consistently reduced prediction error for other genomic prediction studies in wheat (Wallach et al. 2018), maize (Kick and Washburn 2023; Tomura et al. 2025a), and coffee (Nascimento et al. 2024) or reached the equivalent performance to the best multi-kernel RKHS models in wheat and pig traits (Tusell et al. 2014), indicating that the prediction performance of the mean value-based ensemble models in their studies reached the highest or equivalent to that of the best individual prediction model. Hence, both theory and the body of empirical evidence indicate that an ensemble of genomic prediction models may be a more efficient approach than continuous trial-and-error investigation to determine the best individual genomic prediction model for each trait and dataset.

The success of the ensemble approach demonstrated in this study is interpreted in part to be a consequence of the high levels of genetic diversity represented in both datasets (Buckler et al. 2009; Hufford et al. 2012; Chen et al. 2019). The high levels of genetic variation in both NAM experiments contributed to the high levels of diversity in the predicted phenotypes and the estimated genomic marker effects from the individual genomic prediction models. While high diversity in predicted values can improve the performance of the ensemble approach, excessively diverse prediction values may not always lead to further increases in the prediction performance. The level of diversity needs to be constrained within a certain range in relation to the prediction performance of individual prediction models that vary by prediction scenarios (Bian and Chen 2021; Wood et al. 2023). The benefits of such constrained levels of diversity align with the implications of the theoretical framework of the Diversity Prediction Theorem (Page 2018). This framework can be applied to investigate the influence of diverse predicted values [Equation (4) 3rd term] toward the prediction error of the ensemble [Equation (4) 1st term] in relation to the mean predicted errors [Equation (4) 2nd term] of individual prediction models, as was conducted for the TeoNAM and MaizeNAM datasets here.

In addition to the contribution to performance improvement through the higher prediction accuracy and lower prediction error, ensemble approaches also enabled the investigation of the repeatability in the highlighted genomic marker effects included as features in the genomic prediction models. Although the 6 prediction models utilized unique prediction algorithms, ranging from the infinitesimal additive models with shrinkage factors to complex nonlinear prediction mechanisms, the constructed prediction models repeatedly highlighted several genome regions where prior evidence indicates the presence of key genes for control of maize flowering time. This high repeatability showed that prediction models successfully assigned large genomic marker effects within regions containing known genes, interpreted to contribute to increasing their prediction performance. Through combining the genomic prediction models into an ensemble, it is interpreted that the different models capture a larger spectrum of the potential trait genetic variation for the reference population of genotypes in the TeoNAM and MaizeNAM studies. A more comprehensive representation of the trait genetic variation by the ensembles is also interpreted as a contribution to their improved prediction performance. The genome regions with high repeatability across genomic prediction models and the TeoNAM and MaizeNAM datasets provide targets for further investigation to confirm the influences of known genes and potentially discover new genes involved in determining trait genetic variation. The use of a simulation dataset can be a potential method to support further investigations. For example, by predefining QTL regions, the reliability of genome regions highlighted by the ensembles can be quantitatively analyzed by calculating the precision of overlaps between the highlighted and true QTL regions. Reliability can also be investigated in relation to the association with the complexity of the trait genetic architecture embedded in a simulated dataset. Such a comparative analysis can comprehensively assess the reliability of repeatedly highlighted genome regions by ensembles as a future research target.

While the ensemble of diverse individual genomic prediction models can be a promising method for consistent prediction performance improvement with the capture of the trait genetic variation, the operational time to compute the ensemble can be an important factor impacting the practicality of the ensemble for large scale operations in breeding programs. Assuming that input data are preprocessed through imputation and genomic marker filtering, the most time-consuming process can be the development of the input matrix for the ensemble from the predicted phenotype vector of each individual genomic prediction model to apply an efficient mean calculating operation. This process can also be error-prone since the order of data points (RILs) for the predicted phenotypes must be sorted to be identical across all the individual genomic prediction models. This problem can be simplified by the development of a computational pipeline that can automate all tasks involved in the ensemble operation. Using a computational tool such as EasiGP (Tomura et al. 2025b), predicted phenotypes are extracted from user-selected individual genomic prediction models and converted into an input matrix for the ensemble automatically, removing the manual operations once the initial input genomic marker data are provided. The improvement of such ensemble computational tools can be operationalized to help breeders apply the ensemble approach to their large-scale breeding programs within the time requirements for applications.

Given that prediction performance improvement was observed for the simplest ensemble approach (averaging predicted phenotypes with equal weights), which was applied herein to demonstrate the methodology, there is potential for further performance improvement using advanced ensemble design algorithms. One promising approach can be weight optimization. The minimization of the Many-Model error in the Diversity Prediction Theorem (Page 2018) can be employed as one method to optimize the weights, since the Many-Model error represents the prediction error of the ensemble. This and other forms of ensemble optimization are an active area for further research. The integration of prior knowledge into ensembles can be another approach to potentially improve the prediction performance of ensembles. Our investigation of the TeoNAM and MaizeNAM datasets highlighted the effects of several known key flowering-time genes (*ZmCCT9*, *ZmCCT10*, and *ZCN8*) and emphasized the importance of other previously unknown genomic regions. Extracted predictive patterns from interpretable genomic prediction models can be utilized as data-driven prior knowledge to supervise the training of genomic prediction models. Prior knowledge can provide prediction models with external information to integrate natural biophysical laws within their prediction mechanisms (Technow et al. 2015; Messina et al. 2018, 2025; Von Rueden et al. 2021; Diepenbrock et al. 2022; Cooper et al. 2025). Such applications of prior knowledge can mitigate the risk of overfitting prediction models, especially when the training set is small and hence the prediction models cannot be well-trained only using the training set (Schapire et al. 2002). The prediction performance of individual genomic prediction models is expected to increase and, consequently, improve the prediction performance of ensembles of these models.

Overall, our experimental investigation was consistent with previous studies showing that an ensemble of multiple genomic prediction models can improve prediction accuracy, indicating its potential to accelerate genetic gain. In addition to demonstrating an effective approach to achieve high prediction performance across multiple dataset scenarios, the ensemble-based methods can be applied to highlight key genomic regions contributing to the standing genetic variation for traits as demonstrated here for the TeoNAM and MaizeNAM datasets. Such improvements in prediction performance motivate further consideration of ensemble-based prediction methodology for breeding applications. Future studies will explore opportunities to extend the ensemble-average model used in this study, including the assessment of prediction performance improvement by optimizing weightings of models and integrating prior knowledge (Diepenbrock et al. 2022; Messina et al. 2025).

## Data and code availability

The original TeoNAM dataset (Chen et al. 2019) used in this study is located at https://datacommons.cyverse.org/browse/iplant/home/shared/panzea/genotypes/GBS/TeosinteNAM for the genotypes and https://doi.org/10.25386/genetics.9250682 for the phenotypes. The MaizeNAM dataset (Buckler et al. 2009) used in this study is located at https://cbsusrv04.tc.cornell.edu/users/panzea/download.aspx?filegroupid=10 for the genotypes and phenotypes. The computational tool used in this study and the downloaded datasets are located at https://github.com/ShunichiroT/EasiGP.

Supplemental material available at G3 online.

## Supplementary Material

jkag090_Supplementary_Data
