# Supplementary material for: Ensemble-based genomic prediction for maize flowering time improves prediction accuracy and reveals novel insights into trait genetic variation
Source: G3 (Bethesda). 2026 Apr 3;16(6):jkag090. doi: 10.1093/g3journal/jkag090 (PMC13232499; doi:10.1093/g3journal/jkag090)
Supplement: jkag090_Supplementary_Data [file jkag090_supplementary_data.zip › Supplemental_Material_G3-2026-406684.docx]

Supplementary Material

**Table S1:** The final number of recombinant inbred line (RIL) records and genomic markers (SNPs) in each population of the TeoNAM and MaizeNAM dataset.

| Dataset | Population | RIL records | SNP numbers |
| --- | --- | --- | --- |
| TeoNAM | W22TIL01 | 444 | 274 - 322 |
|  | W22TIL03 | 540 | 295 - 342 |
|  | W22TIL11 | 438 | 268 - 314 |
|  | W22TIL14 | 460 | 270 - 320 |
|  | W22TIL25 | 616 | 294 - 341 |
| MaizeNAM | 1 | 194 | 170 - 201 |
|  | 2 | 196 | 177 - 206 |
|  | 3 | 191 | 217 - 251 |
|  | 4 | 196 | 188 - 211 |
|  | 5 | 187 | 209 - 235 |
|  | 6 | 185 | 199 - 222 |
|  | 7 | 193 | 183 - 213 |
|  | 8 | 196 | 206 - 236 |
|  | 9 | 189 | 197 - 225 |
|  | 10 | 192 | 192 - 224 |
|  | 11 | 194 | 186 - 215 |
|  | 12 | 193 | 190 - 218 |
|  | 13 | 126 | 199 - 231 |
|  | 14 | 196 | 182 - 213 |
|  | 15 | 185 | 185 - 210 |
|  | 16 | 194 | 175 - 200 |
|  | 18 | 192 | 176 - 199 |
|  | 19 | 196 | 183 - 206 |
|  | 20 | 188 | 197 - 228 |
|  | 21 | 184 | 190 - 214 |
|  | 22 | 193 | 188 - 215 |
|  | 23 | 181 | 174 - 199 |
|  | 24 | 183 | 183 - 210 |
|  | 25 | 188 | 201 - 227 |
|  | 26 | 187 | 202 - 230 |

**Table S2:** The evaluation of the genomic prediction models (rrBLUP, BayesB, RKHS, RF, SVR, MLP and Ensemble) for the days to anthesis (DTA) and anthesis to silking interval (ASI) traits at the population level in both TeoNAM and MaizeNAM datasets. The prediction performance was measured in a) Pearson correlation and b) mean squared error (MSE). The sign “±” indicates standard error. Values inside brackets represent the ranking of each genomic prediction model under each population-by-trait prediction scenario and the prediction performance improves as the ranking values become smaller.

a)

| Dataset | Population | Trait | Pearson correlation | | | | | | |
| --- | --- | --- | --- | --- | --- | --- | --- | --- | --- |
|  |  |  | rrBLUP | BayesB | RKHS | RF | SVR | MLP | Ensemble |
| TeoNAM | W22TIL01 | DTA | 0.866±0.001(2) | 0.874±0.001(1) | 0.797±0.003(5) | 0.754±0.001(6) | 0.422±0.002(7) | 0.809±0.001(4) | 0.822±0.002(3) |
|  |  | ASI | 0.514±0.002(2) | 0.509±0.002(3) | 0.502±0.002(4) | 0.482±0.002(5) | 0.474±0.002(6) | 0.444±0.002(7) | 0.525±0.002(1) |
|  | W22TIL03 | DTA | 0.864±0.001(2) | 0.874±0.001(1) | 0.824±0.003(3) | 0.731±0.001(6) | 0.444±0.001(7) | 0.816±0.001(5) | 0.820±0.002(4) |
|  |  | ASI | 0.408±0.002(4) | 0.407±0.002(5) | 0.419±0.002(2) | 0.417±0.002(3) | 0.405±0.002(6) | 0.364±0.002(7) | 0.432±0.002(1) |
|  | W22TIL11 | DTA | 0.902±0.001(2) | 0.909±0.001(1) | 0.797±0.005(5) | 0.795±0.001(6) | 0.267±0.002(7) | 0.840±0.001(4) | 0.865±0.001(3) |
|  |  | ASI | 0.489±0.002(5) | 0.493±0.002(4) | 0.516±0.002(1) | 0.474±0.003(6) | 0.499±0.002(3) | 0.436±0.003(7) | 0.508±0.002(2) |
|  | W22TIL14 | DTA | 0.890±0.001(2) | 0.901±0.000(1) | 0.675±0.006(6) | 0.858±0.001(4) | 0.053±0.002(7) | 0.823±0.001(5) | 0.869±0.001(3) |
|  |  | ASI | 0.435±0.002(5) | 0.431±0.002(6) | 0.452±0.002(2) | 0.444±0.002(4) | 0.449±0.002(3) | 0.425±0.002(7) | 0.477±0.002(1) |
|  | W22TIL25 | DTA | 0.866±0.001(2) | 0.877±0.000(1) | 0.820±0.002(4) | 0.777±0.001(6) | 0.375±0.001(7) | 0.803±0.001(5) | 0.832±0.001(3) |
|  |  | ASI | 0.553±0.001(3) | 0.552±0.001(4) | 0.556±0.002(2) | 0.531±0.002(6) | 0.535±0.001(5) | 0.479±0.002(7) | 0.561±0.001(1) |
| MaizeNAM | 1 | DTA | 0.618±0.005(1) | 0.618±0.005(2) | 0.606±0.005(4) | 0.518±0.008(7) | 0.546±0.006(5) | 0.522±0.012(6) | 0.608±0.006(3) |
|  |  | ASI | 0.419±0.008(1) | 0.409±0.008(4) | 0.416±0.008(2) | 0.353±0.008(6) | 0.393±0.008(5) | 0.309±0.009(7) | 0.409±0.008(3) |
|  | 2 | DTA | 0.388±0.008(2) | 0.376±0.008(4) | 0.392±0.008(1) | 0.337±0.009(6) | 0.370±0.008(5) | 0.330±0.009(7) | 0.386±0.008(3) |
|  |  | ASI | 0.518±0.007(3) | 0.520±0.007(2) | 0.517±0.007(4) | 0.458±0.007(6) | 0.491±0.007(5) | 0.429±0.008(7) | 0.524±0.007(1) |
|  | 3 | DTA | 0.687±0.004(3) | 0.691±0.004(2) | 0.675±0.005(4) | 0.578±0.008(7) | 0.609±0.006(6) | 0.641±0.007(5) | 0.691±0.004(1) |
|  |  | ASI | 0.497±0.007(1) | 0.482±0.007(4) | 0.486±0.007(2) | 0.410±0.009(7) | 0.430±0.007(5) | 0.428±0.008(6) | 0.485±0.007(3) |
|  | 4 | DTA | 0.743±0.004(2) | 0.744±0.004(1) | 0.735±0.004(4) | 0.599±0.005(7) | 0.681±0.005(6) | 0.709±0.006(5) | 0.742±0.004(3) |
|  |  | ASI | 0.351±0.008(6) | 0.362±0.008(5) | 0.367±0.008(4) | 0.382±0.008(2) | 0.367±0.008(3) | 0.304±0.009(7) | 0.385±0.008(1) |
|  | 5 | DTA | 0.635±0.005(5) | 0.689±0.005(2) | 0.646±0.005(4) | 0.703±0.005(1) | 0.610±0.005(6) | 0.572±0.007(7) | 0.687±0.005(3) |
|  |  | ASI | 0.470±0.008(2) | 0.483±0.008(1) | 0.466±0.008(3) | 0.386±0.008(6) | 0.439±0.007(5) | 0.383±0.009(7) | 0.460±0.008(4) |
|  | 6 | DTA | 0.662±0.006(3) | 0.665±0.006(1) | 0.633±0.007(4) | 0.523±0.008(7) | 0.548±0.007(6) | 0.596±0.009(5) | 0.664±0.006(2) |
|  |  | ASI | 0.559±0.007(1) | 0.538±0.007(3) | 0.540±0.007(2) | 0.427±0.008(7) | 0.491±0.007(5) | 0.460±0.008(6) | 0.537±0.007(4) |
|  | 7 | DTA | 0.661±0.006(3) | 0.663±0.006(2) | 0.667±0.006(1) | 0.567±0.007(7) | 0.629±0.006(5) | 0.571±0.009(6) | 0.660±0.006(4) |
|  |  | ASI | 0.393±0.008(4) | 0.395±0.008(2) | 0.405±0.008(1) | 0.308±0.009(7) | 0.368±0.008(5) | 0.327±0.009(6) | 0.395±0.008(3) |
|  | 8 | DTA | 0.619±0.005(3) | 0.633±0.006(1) | 0.597±0.005(4) | 0.498±0.008(7) | 0.539±0.006(6) | 0.589±0.007(5) | 0.626±0.005(2) |
|  |  | ASI | 0.499±0.008(4) | 0.499±0.008(5) | 0.509±0.008(1) | 0.418±0.009(7) | 0.503±0.008(2) | 0.425±0.009(6) | 0.500±0.008(3) |
|  | 9 | DTA | 0.641±0.006(2) | 0.635±0.006(4) | 0.637±0.006(3) | 0.581±0.007(7) | 0.587±0.007(6) | 0.591±0.006(5) | 0.650±0.005(1) |
|  |  | ASI | 0.376±0.009(4) | 0.400±0.008(1) | 0.378±0.009(3) | 0.374±0.008(5) | 0.357±0.009(6) | 0.315±0.009(7) | 0.398±0.008(2) |
|  | 10 | DTA | 0.710±0.005(1) | 0.705±0.005(3) | 0.707±0.005(2) | 0.595±0.006(7) | 0.683±0.005(5) | 0.621±0.012(6) | 0.695±0.005(4) |
|  |  | ASI | 0.591±0.006(5) | 0.598±0.006(2) | 0.596±0.006(3) | 0.541±0.006(6) | 0.595±0.006(4) | 0.517±0.008(7) | 0.600±0.006(1) |
|  | 11 | DTA | 0.700±0.007(2) | 0.697±0.007(3) | 0.702±0.007(1) | 0.587±0.008(6) | 0.624±0.007(5) | 0.574±0.013(7) | 0.687±0.007(4) |
|  |  | ASI | 0.451±0.007(4) | 0.458±0.007(2) | 0.455±0.008(3) | 0.429±0.009(6) | 0.438±0.008(5) | 0.390±0.009(7) | 0.459±0.008(1) |
|  | 12 | DTA | 0.696±0.004(4) | 0.714±0.004(1) | 0.708±0.004(3) | 0.658±0.005(6) | 0.681±0.005(5) | 0.630±0.007(7) | 0.710±0.004(2) |
|  |  | ASI | 0.423±0.008(1) | 0.407±0.007(3) | 0.410±0.008(2) | 0.346±0.008(6) | 0.380±0.008(5) | 0.293±0.009(7) | 0.403±0.008(4) |
|  | 13 | DTA | 0.610±0.008(1) | 0.606±0.008(2) | 0.596±0.008(3) | 0.470±0.010(6) | 0.514±0.009(5) | 0.365±0.013(7) | 0.539±0.010(4) |
|  |  | ASI | 0.405±0.009(3) | 0.405±0.010(2) | 0.416±0.009(1) | 0.316±0.010(7) | 0.357±0.009(5) | 0.330±0.011(6) | 0.395±0.009(4) |
|  | 14 | DTA | 0.635±0.006(2) | 0.639±0.006(1) | 0.606±0.006(4) | 0.516±0.007(7) | 0.543±0.007(6) | 0.545±0.010(5) | 0.619±0.006(3) |
|  |  | ASI | 0.517±0.007(4) | 0.526±0.007(1) | 0.522±0.007(2) | 0.486±0.008(6) | 0.515±0.007(5) | 0.417±0.008(7) | 0.522±0.007(3) |
|  | 15 | DTA | 0.564±0.006(3) | 0.574±0.006(2) | 0.561±0.006(4) | 0.558±0.007(5) | 0.516±0.006(6) | 0.513±0.008(7) | 0.578±0.006(1) |
|  |  | ASI | 0.448±0.008(2) | 0.444±0.008(4) | 0.447±0.008(3) | 0.389±0.009(7) | 0.407±0.008(5) | 0.391±0.009(6) | 0.454±0.008(1) |
|  | 16 | DTA | 0.725±0.004(2) | 0.722±0.004(3) | 0.722±0.005(4) | 0.608±0.007(7) | 0.627±0.006(6) | 0.657±0.008(5) | 0.725±0.005(1) |
|  |  | ASI | 0.330±0.008(2) | 0.344±0.008(1) | 0.321±0.008(5) | 0.325±0.008(4) | 0.296±0.008(6) | 0.215±0.008(7) | 0.325±0.007(3) |
|  | 18 | DTA | 0.688±0.005(3) | 0.692±0.005(2) | 0.682±0.005(4) | 0.580±0.008(7) | 0.625±0.006(6) | 0.660±0.006(5) | 0.693±0.005(1) |
|  |  | ASI | 0.499±0.006(3) | 0.504±0.007(1) | 0.501±0.006(2) | 0.405±0.008(6) | 0.471±0.007(5) | 0.389±0.007(7) | 0.495±0.006(4) |
|  | 19 | DTA | 0.494±0.007(4) | 0.510±0.007(2) | 0.512±0.007(1) | 0.492±0.008(5) | 0.478±0.008(6) | 0.371±0.009(7) | 0.496±0.007(3) |
|  |  | ASI | 0.592±0.005(2) | 0.580±0.005(5) | 0.589±0.005(3) | 0.516±0.007(7) | 0.583±0.006(4) | 0.555±0.006(6) | 0.600±0.005(1) |
|  | 20 | DTA | 0.581±0.006(4) | 0.592±0.006(1) | 0.588±0.006(3) | 0.510±0.007(7) | 0.534±0.007(5) | 0.514±0.009(6) | 0.589±0.007(2) |
|  |  | ASI | 0.354±0.007(3) | 0.355±0.007(2) | 0.350±0.007(4) | 0.342±0.009(5) | 0.320±0.007(6) | 0.296±0.008(7) | 0.358±0.007(1) |
|  | 21 | DTA | 0.665±0.006(2) | 0.666±0.006(1) | 0.661±0.006(3) | 0.555±0.007(7) | 0.612±0.006(5) | 0.570±0.011(6) | 0.658±0.007(4) |
|  |  | ASI | 0.413±0.008(1) | 0.403±0.008(2) | 0.398±0.008(4) | 0.282±0.010(7) | 0.346±0.009(5) | 0.333±0.010(6) | 0.400±0.008(3) |
|  | 22 | DTA | 0.500±0.008(2) | 0.496±0.007(3) | 0.501±0.007(1) | 0.424±0.008(6) | 0.439±0.008(5) | 0.347±0.015(7) | 0.466±0.008(4) |
|  |  | ASI | 0.621±0.006(1) | 0.615±0.006(2) | 0.601±0.006(4) | 0.477±0.008(7) | 0.557±0.007(5) | 0.503±0.007(6) | 0.608±0.006(3) |
|  | 23 | DTA | 0.623±0.006(2) | 0.647±0.006(1) | 0.597±0.006(4) | 0.528±0.007(7) | 0.547±0.006(5) | 0.544±0.009(6) | 0.622±0.006(3) |
|  |  | ASI | 0.480±0.008(2) | 0.478±0.008(3) | 0.487±0.008(1) | 0.424±0.009(6) | 0.444±0.009(5) | 0.422±0.008(7) | 0.477±0.008(4) |
|  | 24 | DTA | 0.668±0.005(1) | 0.651±0.005(4) | 0.663±0.005(2) | 0.582±0.007(6) | 0.586±0.006(5) | 0.516±0.014(7) | 0.657±0.006(3) |
|  |  | ASI | 0.434±0.008(3) | 0.442±0.008(1) | 0.410±0.008(4) | 0.398±0.008(5) | 0.387±0.008(6) | 0.377±0.008(7) | 0.436±0.007(2) |
|  | 25 | DTA | 0.399±0.007(5) | 0.403±0.008(4) | 0.406±0.007(3) | 0.391±0.008(6) | 0.440±0.007(1) | 0.353±0.009(7) | 0.419±0.007(2) |
|  |  | ASI | 0.555±0.006(3) | 0.542±0.006(5) | 0.562±0.006(2) | 0.473±0.007(7) | 0.549±0.006(4) | 0.510±0.007(6) | 0.562±0.006(1) |
|  | 26 | DTA | 0.638±0.006(2) | 0.640±0.005(1) | 0.628±0.006(4) | 0.523±0.007(7) | 0.587±0.007(5) | 0.548±0.008(6) | 0.628±0.005(3) |
|  |  | ASI | 0.252±0.008(5) | 0.253±0.008(3) | 0.267±0.008(1) | 0.200±0.009(7) | 0.253±0.008(4) | 0.232±0.009(6) | 0.255±0.008(2) |

b)

| Dataset | Population | Trait | MSE | | | | | | |
| --- | --- | --- | --- | --- | --- | --- | --- | --- | --- |
|  |  |  | rrBLUP | BayesB | RKHS | RF | SVR | MLP | Ensemble |
| TeoNAM | W22TIL01 | DTA | 8.032±0.004(2) | 7.537±0.004(1) | 11.993±0.004(5) | 13.984±0.038(6) | 28.004±0.017(7) | 11.693±0.239(4) | 10.531±0.004(3) |
|  |  | ASI | 3.405±0.007(2) | 3.419±0.004(3) | 3.469±0.004(4) | 3.575±0.062(5) | 3.657±0.018(6) | 4.201±0.205(7) | 3.342±0.007(1) |
|  | W22TIL03 | DTA | 8.267±0.008(2) | 7.736±0.005(1) | 10.788±0.006(3) | 15.309±0.015(6) | 27.971±0.021(7) | 11.599±0.627(5) | 10.864±0.008(4) |
|  |  | ASI | 4.921±0.004(4) | 4.926±0.019(5) | 4.870±0.020(2) | 4.906±0.039(3) | 5.024±0.025(6) | 6.026±0.324(7) | 4.793±0.004(1) |
|  | W22TIL11 | DTA | 5.938±0.007(2) | 5.463±0.009(1) | 11.860±0.010(6) | 11.596±0.019(5) | 29.347±0.019(7) | 10.435±0.228(4) | 8.550±0.007(3) |
|  |  | ASI | 3.310±0.008(4) | 3.305±0.012(3) | 3.225±0.012(2) | 3.418±0.016(6) | 3.385±0.054(5) | 3.983±0.714(7) | 3.223±0.008(1) |
|  | W22TIL14 | DTA | 12.897±0.017(2) | 11.604±0.004(1) | 35.802±0.004(6) | 16.369±0.031(3) | 61.753±0.049(7) | 22.279±0.223(5) | 17.077±0.017(4) |
|  |  | ASI | 6.010±0.003(4) | 6.045±0.006(5) | 5.903±0.006(2) | 5.970±0.053(3) | 6.213±0.045(6) | 6.726±1.269(7) | 5.717±0.003(1) |
|  | W22TIL25 | DTA | 8.728±0.008(2) | 8.074±0.005(1) | 11.857±0.006(4) | 13.922±0.062(6) | 31.094±0.063(7) | 13.259±0.209(5) | 10.974±0.008(3) |
|  |  | ASI | 4.734±0.004(2) | 4.755±0.005(4) | 4.748±0.006(3) | 4.913±0.061(5) | 5.079±0.045(6) | 5.770±0.201(7) | 4.679±0.004(1) |
| MaizeNAM | 1 | DTA | 2.333±0.034(2) | 2.360±0.034(3) | 2.439±0.036(4) | 2.905±0.045(6) | 1.696±0.046(1) | 8.328±0.879(7) | 2.554±0.049(5) |
|  |  | ASI | 0.804±0.011(2) | 0.819±0.011(5) | 0.807±0.010(3) | 0.859±0.010(6) | 0.335±0.010(1) | 0.966±0.014(7) | 0.816±0.010(4) |
|  | 2 | DTA | 3.365±0.049(3) | 3.414±0.050(4) | 3.349±0.050(2) | 3.505±0.051(6) | 2.112±0.053(1) | 8.646±0.571(7) | 3.436±0.053(5) |
|  |  | ASI | 0.733±0.011(3) | 0.736±0.011(4) | 0.737±0.011(5) | 0.791±0.011(6) | 0.282±0.011(1) | 0.844±0.013(7) | 0.732±0.011(2) |
|  | 3 | DTA | 5.130±0.077(2) | 5.083±0.081(1) | 5.574±0.091(4) | 6.557±0.115(6) | 6.531±0.127(5) | 10.259±0.459(7) | 5.366±0.095(3) |
|  |  | ASI | 0.948±0.012(2) | 0.967±0.012(3) | 0.968±0.012(4) | 1.074±0.014(7) | 0.470±0.014(1) | 1.070±0.016(6) | 0.970±0.013(5) |
|  | 4 | DTA | 3.993±0.061(1) | 4.031±0.061(2) | 4.291±0.064(3) | 6.059±0.077(6) | 5.262±0.083(5) | 8.731±0.544(7) | 4.338±0.070(4) |
|  |  | ASI | 1.585±0.035(6) | 1.573±0.034(5) | 1.553±0.035(3) | 1.559±0.034(4) | 0.818±0.036(1) | 1.736±0.038(7) | 1.536±0.035(2) |
|  | 5 | DTA | 6.499±0.091(4) | 5.746±0.087(2) | 6.537±0.095(5) | 5.681±0.089(1) | 7.878±0.161(6) | 15.026±0.814(7) | 6.104±0.100(3) |
|  |  | ASI | 1.286±0.019(3) | 1.279±0.019(2) | 1.289±0.019(4) | 1.390±0.020(6) | 0.687±0.020(1) | 1.490±0.025(7) | 1.294±0.019(5) |
|  | 6 | DTA | 3.351±0.051(3) | 3.329±0.050(2) | 3.649±0.053(5) | 4.410±0.054(6) | 3.291±0.062(1) | 7.488±0.378(7) | 3.481±0.051(4) |
|  |  | ASI | 1.071±0.022(2) | 1.109±0.022(4) | 1.106±0.023(3) | 1.273±0.023(7) | 0.637±0.024(1) | 1.256±0.024(6) | 1.122±0.023(5) |
|  | 7 | DTA | 3.366±0.048(3) | 3.325±0.049(2) | 3.381±0.049(4) | 4.136±0.059(6) | 3.099±0.064(1) | 7.421±0.293(7) | 3.475±0.052(5) |
|  |  | ASI | 0.856±0.010(4) | 0.856±0.010(5) | 0.840±0.010(2) | 0.917±0.011(6) | 0.320±0.011(1) | 0.969±0.013(7) | 0.850±0.010(3) |
|  | 8 | DTA | 4.565±0.072(2) | 4.481±0.074(1) | 4.854±0.074(5) | 5.789±0.084(6) | 4.730±0.091(4) | 10.543±0.583(7) | 4.661±0.082(3) |
|  |  | ASI | 0.981±0.014(3) | 0.995±0.014(5) | 0.970±0.014(2) | 1.072±0.013(6) | 0.463±0.013(1) | 1.130±0.017(7) | 0.984±0.013(4) |
|  | 9 | DTA | 2.871±0.050(2) | 2.873±0.050(4) | 2.963±0.053(5) | 3.305±0.053(6) | 2.441±0.062(1) | 7.464±0.395(7) | 2.872±0.052(3) |
|  |  | ASI | 0.947±0.012(6) | 0.920±0.011(3) | 0.933±0.012(4) | 0.937±0.011(5) | 0.426±0.013(1) | 1.064±0.015(7) | 0.919±0.012(2) |
|  | 10 | DTA | 2.701±0.039(2) | 2.748±0.041(3) | 2.823±0.039(4) | 3.703±0.047(6) | 2.564±0.046(1) | 7.784±0.400(7) | 2.984±0.046(5) |
|  |  | ASI | 0.819±0.012(4) | 0.806±0.012(2) | 0.825±0.012(5) | 0.891±0.014(6) | 0.374±0.014(1) | 0.963±0.015(7) | 0.815±0.013(3) |
|  | 11 | DTA | 3.048±0.040(1) | 3.053±0.040(2) | 3.150±0.044(4) | 3.955±0.055(6) | 3.088±0.069(3) | 9.112±0.729(7) | 3.327±0.055(5) |
|  |  | ASI | 0.853±0.012(4) | 0.851±0.012(3) | 0.857±0.012(5) | 0.888±0.013(6) | 0.325±0.012(1) | 0.954±0.014(7) | 0.845±0.012(2) |
|  | 12 | DTA | 4.700±0.065(2) | 4.553±0.065(1) | 4.739±0.069(4) | 5.199±0.076(5) | 5.299±0.097(6) | 9.709±0.362(7) | 4.721±0.068(3) |
|  |  | ASI | 1.055±0.015(2) | 1.073±0.015(5) | 1.066±0.015(3) | 1.132±0.015(6) | 0.526±0.016(1) | 1.242±0.019(7) | 1.069±0.015(4) |
|  | 13 | DTA | 3.913±0.064(2) | 3.971±0.065(3) | 4.254±0.071(4) | 4.859±0.074(6) | 3.654±0.077(1) | 35.210±1.792(7) | 4.622±0.095(5) |
|  |  | ASI | 1.089±0.017(3) | 1.106±0.017(5) | 1.089±0.018(4) | 1.156±0.017(6) | 0.505±0.019(1) | 1.218±0.021(7) | 1.088±0.018(2) |
|  | 14 | DTA | 2.183±0.030(2) | 2.192±0.031(3) | 2.349±0.031(4) | 2.832±0.036(6) | 1.585±0.034(1) | 6.314±0.529(7) | 2.350±0.036(5) |
|  |  | ASI | 0.784±0.009(4) | 0.778±0.010(2) | 0.783±0.009(3) | 0.834±0.010(6) | 0.352±0.009(1) | 0.937±0.012(7) | 0.785±0.009(5) |
|  | 15 | DTA | 3.768±0.057(4) | 3.682±0.056(2) | 3.772±0.062(5) | 3.877±0.060(6) | 2.919±0.084(1) | 8.639±0.487(7) | 3.752±0.066(3) |
|  |  | ASI | 0.979±0.014(3) | 0.987±0.014(5) | 0.981±0.014(4) | 1.061±0.014(6) | 0.503±0.016(1) | 1.064±0.016(7) | 0.975±0.014(2) |
|  | 16 | DTA | 2.415±0.036(2) | 2.388±0.037(1) | 2.603±0.045(4) | 3.271±0.055(6) | 2.488±0.058(3) | 7.801±0.652(7) | 2.641±0.051(5) |
|  |  | ASI | 1.027±0.019(5) | 1.023±0.019(2) | 1.027±0.019(4) | 1.039±0.019(6) | 0.427±0.020(1) | 1.216±0.023(7) | 1.025±0.019(3) |
|  | 18 | DTA | 3.915±0.059(2) | 3.864±0.059(1) | 4.126±0.068(4) | 5.253±0.080(6) | 4.373±0.086(5) | 8.291±0.410(7) | 4.054±0.065(3) |
|  |  | ASI | 1.707±0.025(2) | 1.717±0.025(3) | 1.720±0.025(4) | 1.944±0.027(6) | 1.189±0.028(1) | 2.026±0.032(7) | 1.739±0.026(5) |
|  | 19 | DTA | 2.424±0.030(4) | 2.384±0.030(2) | 2.397±0.029(3) | 2.468±0.030(5) | 1.249±0.033(1) | 6.291±0.417(7) | 2.486±0.032(6) |
|  |  | ASI | 0.687±0.008(3) | 0.704±0.008(5) | 0.687±0.008(4) | 0.782±0.010(7) | 0.289±0.009(1) | 0.761±0.018(6) | 0.683±0.008(2) |
|  | 20 | DTA | 3.330±0.051(3) | 3.307±0.051(2) | 3.348±0.052(4) | 3.747±0.056(6) | 2.699±0.056(1) | 9.505±0.540(7) | 3.416±0.058(5) |
|  |  | ASI | 1.220±0.015(4) | 1.223±0.015(6) | 1.214±0.015(3) | 1.221±0.017(5) | 0.627±0.016(1) | 1.378±0.018(7) | 1.207±0.015(2) |
|  | 21 | DTA | 1.970±0.032(3) | 1.946±0.031(2) | 2.034±0.032(4) | 2.543±0.034(6) | 1.413±0.037(1) | 5.903±0.352(7) | 2.073±0.037(5) |
|  |  | ASI | 0.784±0.012(2) | 0.791±0.013(4) | 0.790±0.013(3) | 0.857±0.013(6) | 0.271±0.013(1) | 0.908±0.015(7) | 0.794±0.013(5) |
|  | 22 | DTA | 2.339±0.033(3) | 2.333±0.034(2) | 2.351±0.034(4) | 2.560±0.032(6) | 1.301±0.039(1) | 5.788±0.316(7) | 2.421±0.038(5) |
|  |  | ASI | 0.920±0.015(3) | 0.919±0.014(2) | 0.973±0.015(4) | 1.178±0.015(7) | 0.563±0.016(1) | 1.115±0.016(6) | 0.975±0.015(5) |
|  | 23 | DTA | 2.951±0.043(3) | 2.803±0.043(2) | 3.150±0.044(5) | 3.535±0.048(6) | 2.492±0.051(1) | 7.274±0.524(7) | 3.067±0.049(4) |
|  |  | ASI | 1.184±0.014(3) | 1.194±0.015(5) | 1.182±0.015(2) | 1.293±0.017(6) | 0.635±0.017(1) | 1.337±0.018(7) | 1.188±0.015(4) |
|  | 24 | DTA | 3.800±0.058(1) | 3.946±0.058(2) | 3.994±0.064(3) | 4.665±0.069(6) | 4.074±0.082(4) | 9.252±0.533(7) | 4.117±0.072(5) |
|  |  | ASI | 0.887±0.013(3) | 0.891±0.013(4) | 0.907±0.013(5) | 0.925±0.014(6) | 0.358±0.013(1) | 0.968±0.015(7) | 0.881±0.013(2) |
|  | 25 | DTA | 4.971±0.093(5) | 4.948±0.093(4) | 4.929±0.092(2) | 4.994±0.086(6) | 3.636±0.095(1) | 10.508±0.480(7) | 4.941±0.097(3) |
|  |  | ASI | 1.519±0.021(3) | 1.549±0.020(5) | 1.505±0.021(2) | 1.759±0.022(7) | 1.054±0.023(1) | 1.678±0.026(6) | 1.524±0.021(4) |
|  | 26 | DTA | 3.841±0.065(2) | 3.825±0.063(1) | 4.034±0.074(4) | 4.809±0.081(6) | 3.954±0.087(3) | 12.115±0.740(7) | 4.081±0.073(5) |
|  |  | ASI | 2.073±0.024(5) | 2.066±0.025(4) | 2.026±0.024(2) | 2.093±0.025(6) | 1.338±0.026(1) | 2.310±0.033(7) | 2.036±0.025(3) |

**Table S3:** Pearson correlation of each pair of individual genomic prediction models at the level of the predicted phenotypes (the top right triangle) and the genomic marker effects (the bottom left triangle) for the days to anthesis (DTA) trait in the a) TeoNAM and b) MaizeNAM datasets. Each value represents the correlation of its corresponding subplot in Figure S7.

a)

|  | **rrBLUP** | **BayesB** | **RKHS** | **RF** | **SVR** | **MLP** |
| --- | --- | --- | --- | --- | --- | --- |
| **rrBLUP** |  | 0.976 | 0.807 | 0.823 | 0.408 | 0.525 |
| **BayesB** | 0.738 |  | 0.762 | 0.884 | 0.381 | 0.512 |
| **RKHS** | 0.396 | 0.497 |  | 0.569 | 0.619 | 0.452 |
| **RF** | 0.355 | 0.629 | 0.406 |  | 0.310 | 0.408 |
| **SVR** | 0.143 | 0.258 | 0.559 | 0.332 |  | 0.231 |
| **MLP** | 0.062 | 0.062 | 0.071 | 0.036 | 0.062 |  |

b)

|  | **rrBLUP** | **BayesB** | **RKHS** | **RF** | **SVR** | **MLP** |
| --- | --- | --- | --- | --- | --- | --- |
| **rrBLUP** |  | 0.996 | 0.992 | 0.958 | 0.944 | 0.807 |
| **BayesB** | 0.866 |  | 0.988 | 0.962 | 0.940 | 0.804 |
| **RKHS** | 0.075 | 0.061 |  | 0.975 | 0.972 | 0.787 |
| **RF** | 0.452 | 0.629 | 0.048 |  | 0.970 | 0.749 |
| **SVR** | 0.508 | 0.541 | 0.066 | 0.647 |  | 0.726 |
| **MLP** | 0.457 | 0.437 | 0.029 | 0.209 | 0.243 |  |

**Table S4:** Pearson correlation of each individual genomic prediction model pair at the level of the predicted phenotypes (the top right triangle) and the genomic marker effects (the bottom left triangle) for the anthesis to silking interval (ASI) trait in the a) TeoNAM and b) MaizeNAM datasets. Each value represents the correlation of its corresponding subplot in Figure S8.

a)

|  | **rrBLUP** | **BayesB** | **RKHS** | **RF** | **SVR** | **MLP** |
| --- | --- | --- | --- | --- | --- | --- |
| **rrBLUP** |  | 0.991 | 0.942 | 0.839 | 0.850 | 0.661 |
| **BayesB** | 0.899 |  | 0.931 | 0.841 | 0.842 | 0.654 |
| **RKHS** | 0.777 | 0.716 |  | 0.927 | 0.896 | 0.715 |
| **RF** | 0.437 | 0.561 | 0.417 |  | 0.845 | 0.718 |
| **SVR** | 0.532 | 0.508 | 0.724 | 0.397 |  | 0.621 |
| **MLP** | 0.356 | 0.341 | 0.353 | 0.193 | 0.267 |  |

b)

|  | **rrBLUP** | **BayesB** | **RKHS** | **RF** | **SVR** | **MLP** |
| --- | --- | --- | --- | --- | --- | --- |
| **rrBLUP** |  | 0.991 | 0.985 | 0.895 | 0.923 | 0.874 |
| **BayesB** | 0.902 |  | 0.979 | 0.913 | 0.921 | 0.867 |
| **RKHS** | 0.062 | 0.055 |  | 0.928 | 0.965 | 0.883 |
| **RF** | 0.499 | 0.643 | 0.047 |  | 0.933 | 0.820 |
| **SVR** | 0.584 | 0.652 | 0.058 | 0.698 |  | 0.845 |
| **MLP** | 0.712 | 0.666 | 0.053 | 0.425 | 0.542 |  |


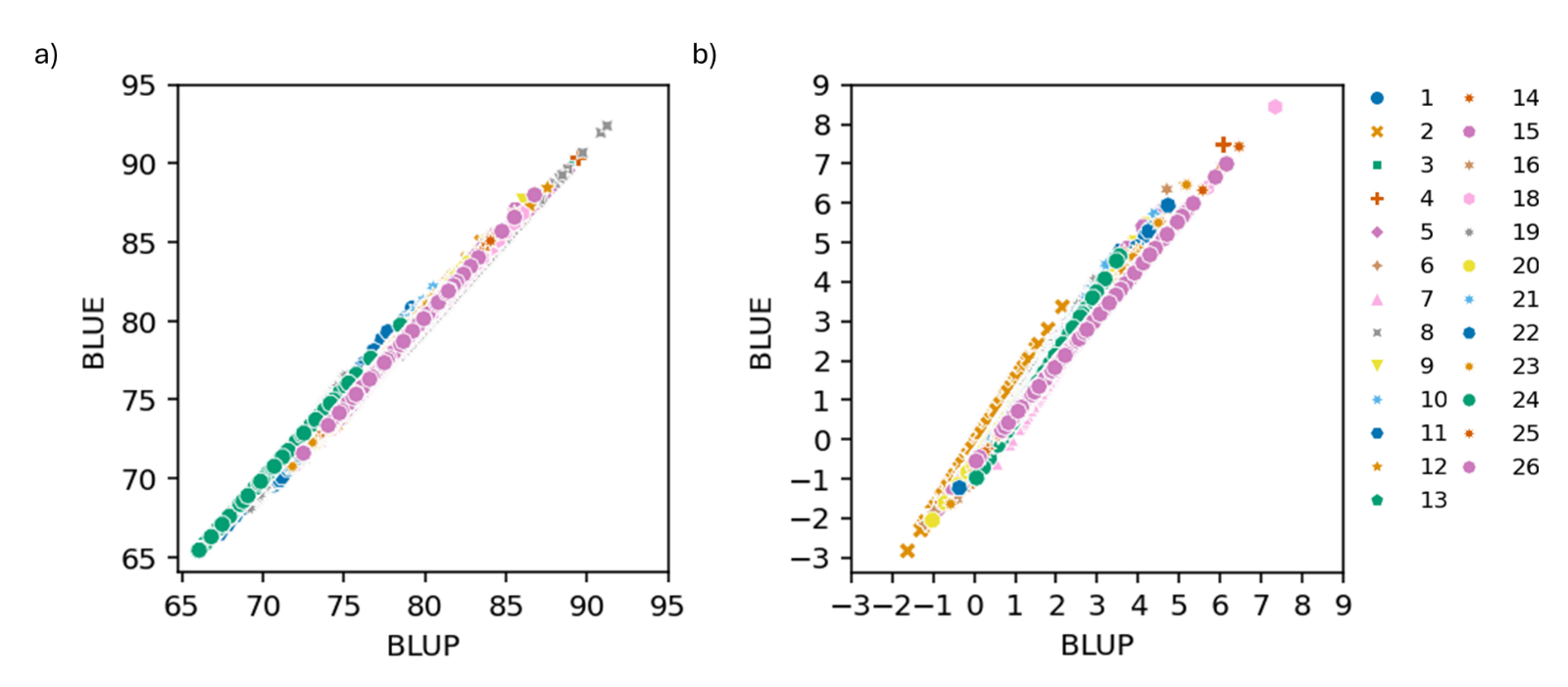


**Fig. S1**: Scatter plots comparing the best linear unbiased predictors (BLUP) and the best linear unbiased estimates (BLUE) at the population level for a) the days to anthesis (DTA) and b) anthesis to silking interval (ASI) traits in the MaizeNAM dataset. The numbers in the legend indicate the population number in the MaizeNAM dataset, showing 25 populations in total.


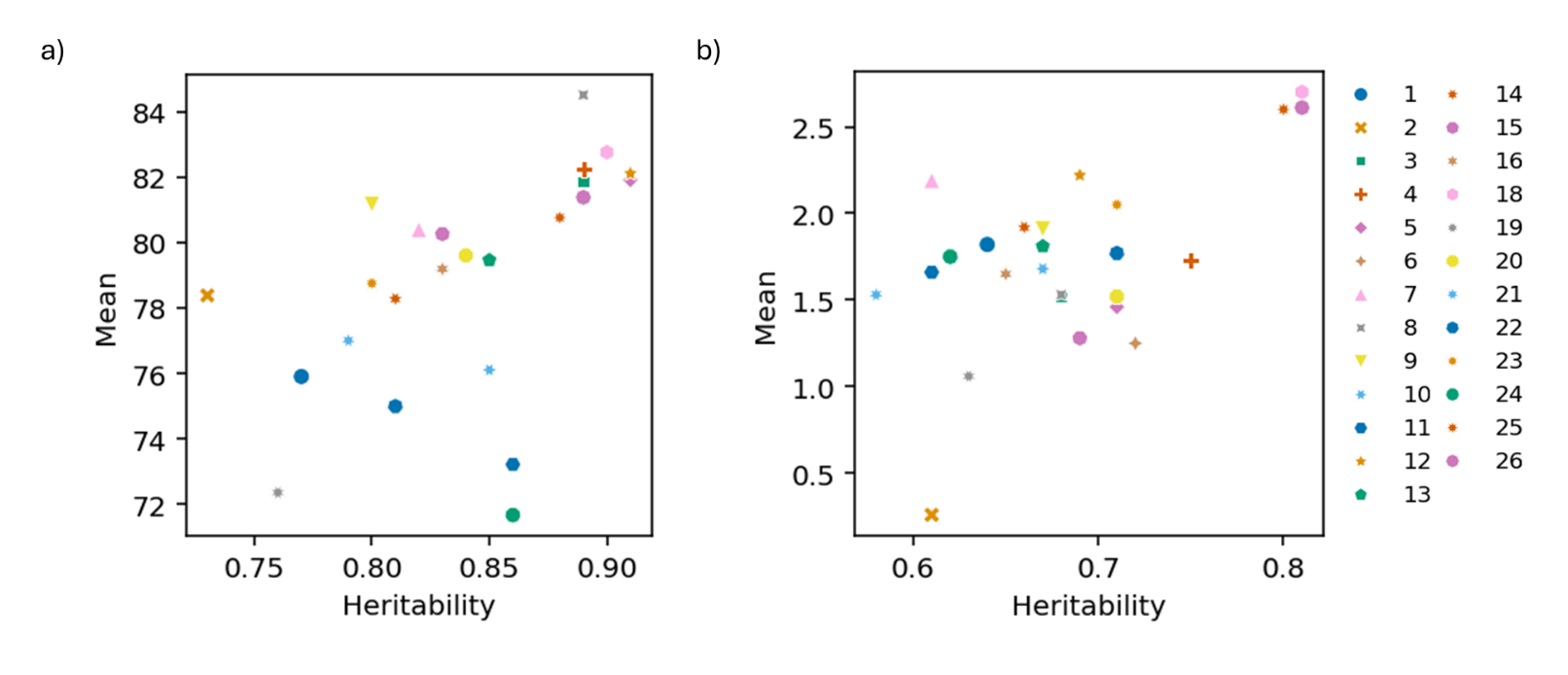


**Fig. S2**: Scatter plots comparing heritability and mean phenotype value in each population for a) the days to anthesis (DTA) and b) anthesis to silking interval (ASI) traits in the MaizeNAM dataset. The numbers in the legend indicate the population number in the MaizeNAM dataset, showing 25 populations in total.


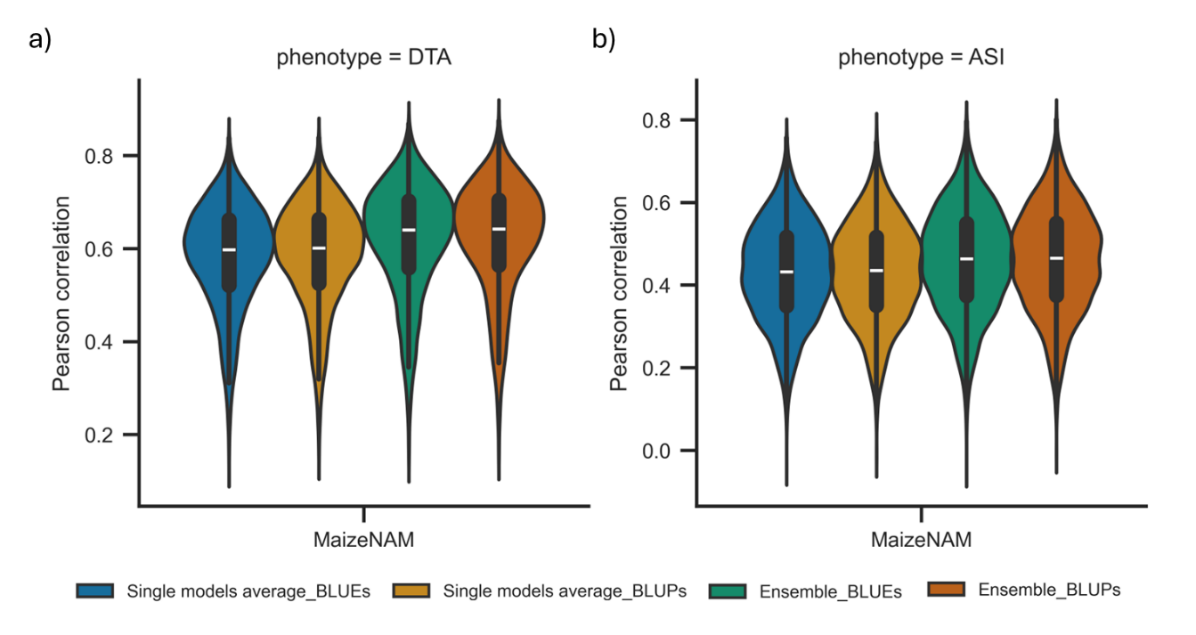


**Fig. S3**: The comparison of Pearson correlation for predicting the best linear unbiased estimates (BLUEs) and the best linear unbiased predictions (BLUPs) between the mean of the 6 individual genomic prediction models (rrBLUP, BayesB, RKHS, RF, SVR and MLP) (Single models average) and the ensemble-average model (Ensemble) in the MaizeNAM dataset. a) the days to anthesis (DTA) and b) anthesis to silking interval (ASI) traits were targeted for the performance comparison. The prediction performance was measured in 3,750 prediction scenarios for each trait. The prediction scenarios were generated by the combination of the 3 training-test ratios (0.8-0.2, 0.65-0.35 and 0.5-0.5), populations and sampling numbers. The width of the violins represents the distribution of performance metrics. The white horizontal lines on the black box plots show the median value for each metric. The whiskers extend 1.5 times the interquartile range.


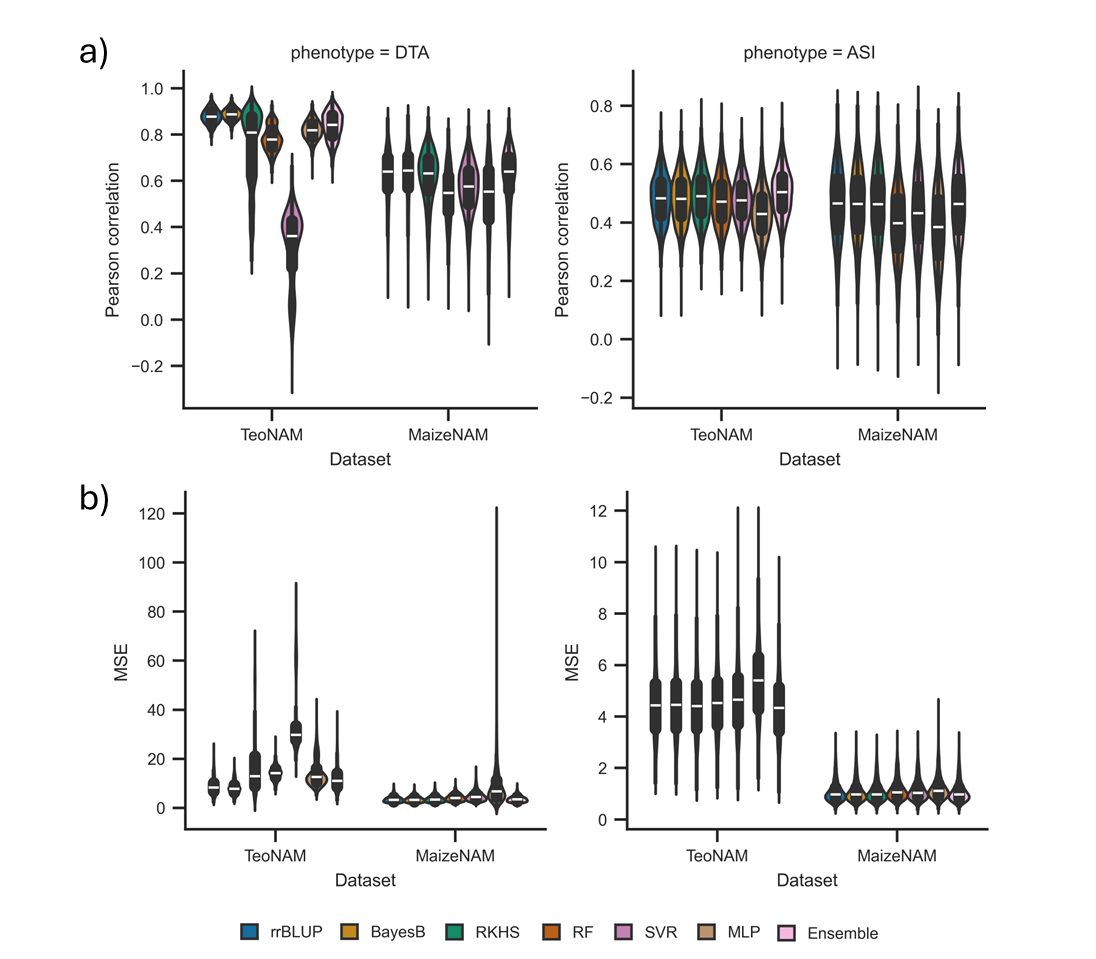


**Fig. S4:** The comparison of prediction performance between the 6 individual genomic prediction models (rrBLUP, BayesB, RKHS, RF, SVR and MLP) and the ensemble-average model (Ensemble) for the days to anthesis (DTA) and anthesis to silking interval (ASI) traits in the TeoNAM and MaizeNAM datasets. The prediction performance was measured using the 2 metrics: a) Pearson correlation and b) mean squared error (MSE). The prediction performance was measured in 7,500 prediction scenarios for the TeoNAM dataset and 3,750 prediction scenarios for the MaizeNAM dataset for each trait. The prediction scenarios were generated by the combination of the 3 training-test ratios (0.8-0.2, 0.65-0.35 and 0.5-0.5), populations and sampling numbers. The width of the violins represents the distribution of performance metrics. The white horizontal lines on the black box plots show the median value for each metric. The whiskers extend 1.5 times the interquartile range.


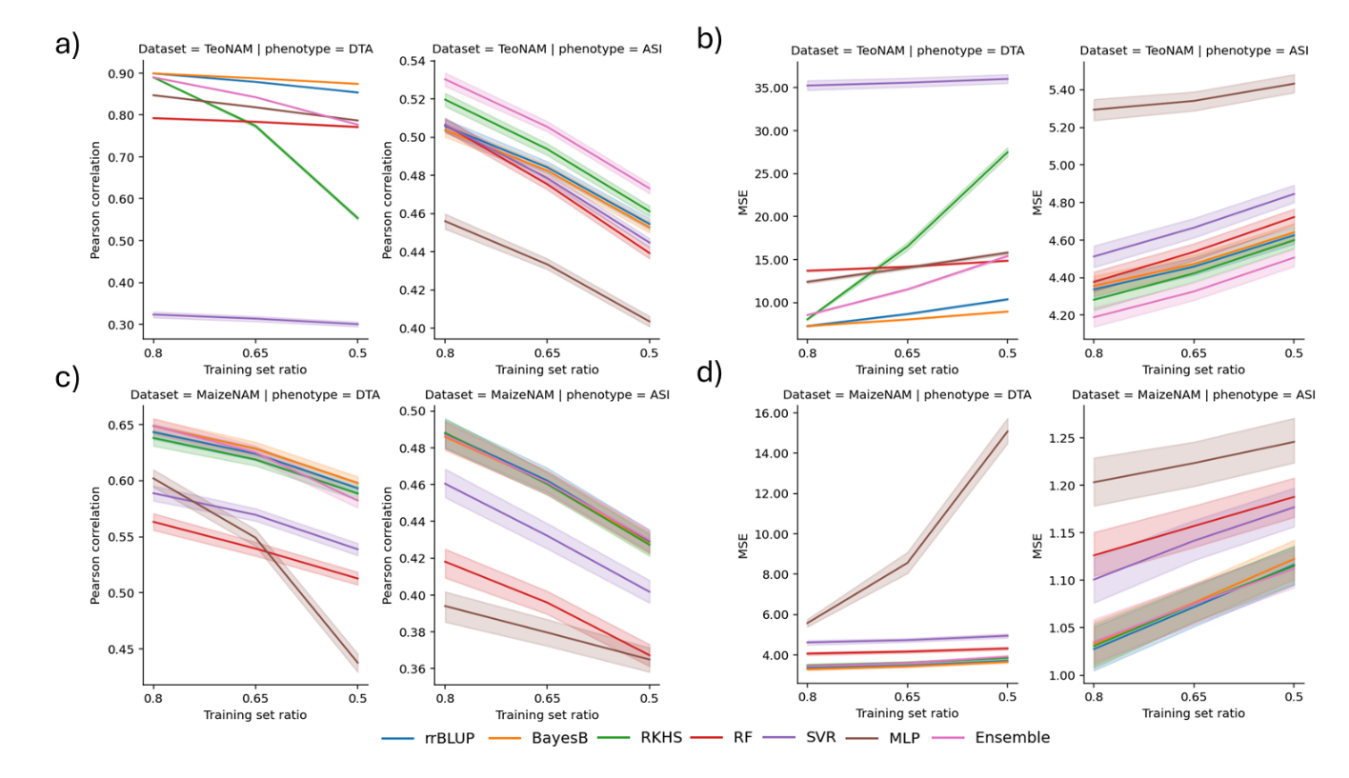


**Fig. S5**: The performance metric transition of the genomic prediction models (rrBLUP, BayesB, RKHS, RF, SVR, MLP and Ensemble) over the ratio reduction of training set for the days to anthesis (DTA) and anthesis to silking interval (ASI) traits: a) Pearson correlation and b) mean squared error (MSE) in the TeoNAM dataset and c) Pearson correlation and d) MSE in the MaizeNAM dataset. In each subplot, the x-axis represents the ratio of the training set whereas the y-axis indicates corresponding metric values. Intervals surrounding each line illustrates 95% the confidence interval.


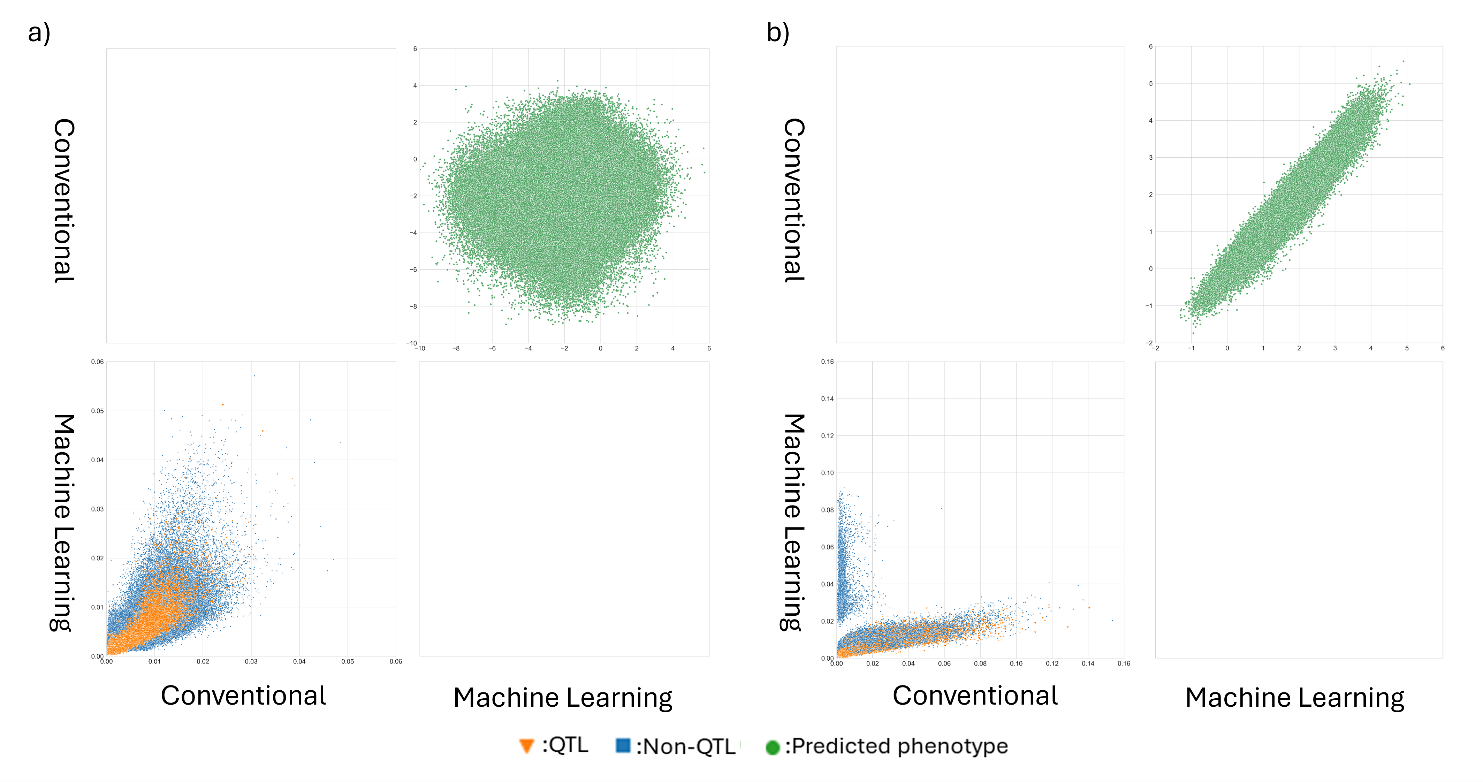


**Fig. S6:** Pairwise comparisons of the conventional (rrBLUP, BayesB and RKHS) and machine learning models (RF, SVR and MLP) for the anthesis to silking interval (ASI) trait across all the prediction scenarios for the a) TeoNAM (7,500 prediction scenarios) and b) MaizeNAM (3,750 prediction scenarios) datasets. The prediction scenarios were generated by the combination of the 3 training-test ratios (0.8-0.2, 0.65-0.35 and 0.5-0.5), populations and sampling numbers. The genomic prediction model groups were compared for mean predicted phenotypes (top right triangle) and mean normalized genomic marker effects (the bottom left triangle), calculated within each prediction model category. The green dots represent a pair of predicted phenotypes of RIL samples in the test sets for each prediction scenario. The blue squares and orange triangles represent a pair of extracted genomic marker effects from each genomic marker in each sample scenario that were identified as non-QTL and QTL markers, respectively, by Chen et al. (2019) for the TeoNAM dataset and Buckler et al. (2009) for the MaizeNAM dataset.


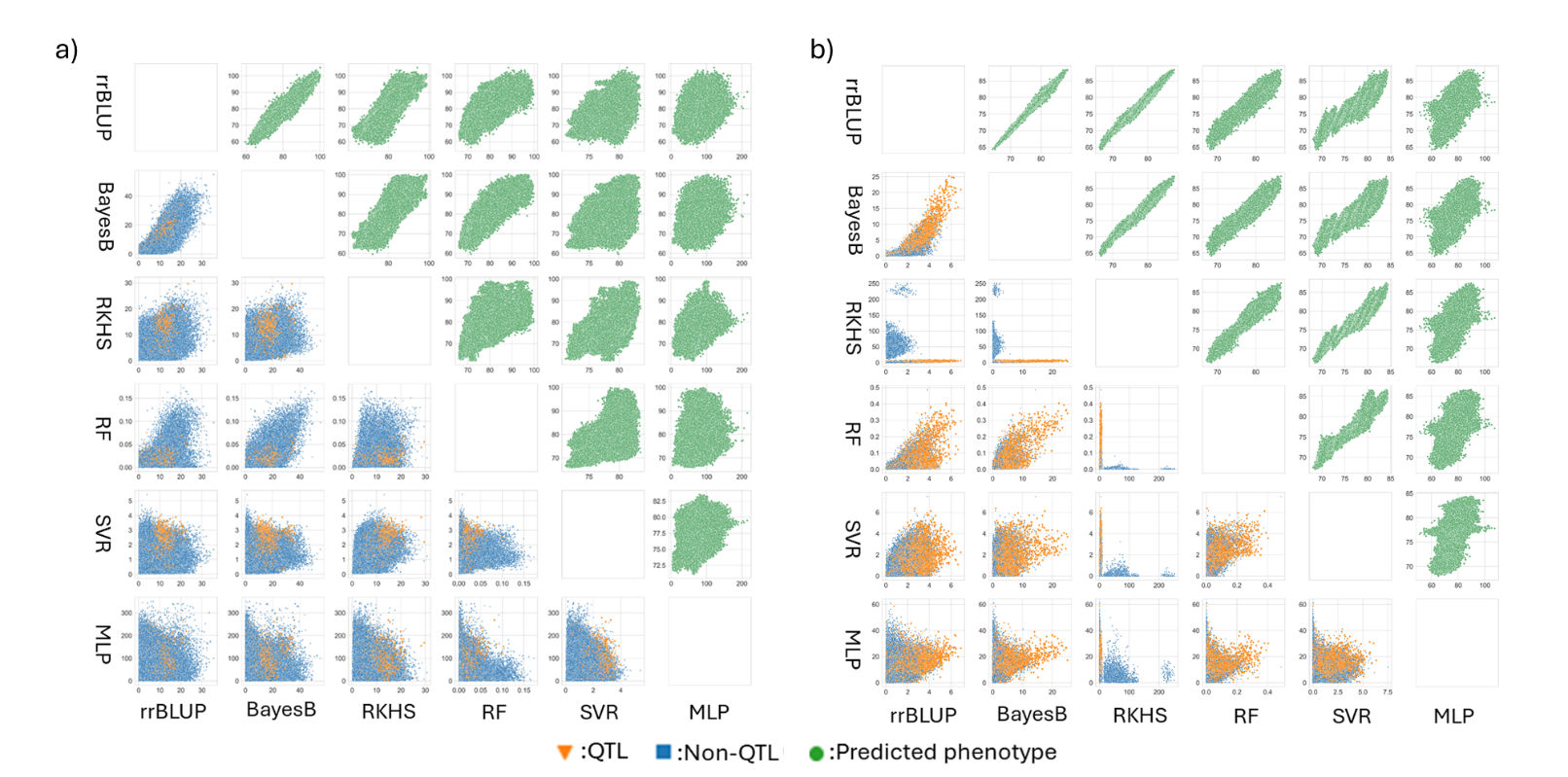


**Fig. S7:** Pairwise comparisons of the individual genomic prediction models (rrBLUP, BayesB, RKHS, RF, SVR and MLP) for the days to anthesis (DTA) trait across all the prediction scenarios for the a) TeoNAM (7,500 prediction scenarios) and b) MaizeNAM (3,750 prediction scenarios) datasets. The prediction scenarios were generated by the combination of the 3 training-test ratios (0.8-0.2, 0.65-0.35 and 0.5-0.5), populations and sampling numbers. The genomic prediction models were compared at the level of predicted phenotypes (top right triangle) and genomic marker effects (the bottom left triangle). The green dots represent a pair of predicted phenotypes of RIL samples in the test sets for each prediction scenario. The blue squares and orange triangles represent a pair of extracted genomic marker effects from each genomic marker in each sample scenario that were identified as non-QTL and QTL markers, respectively, by Chen et al. (2019) for the TeoNAM dataset and Buckler et al. (2009) for the MaizeNAM dataset.


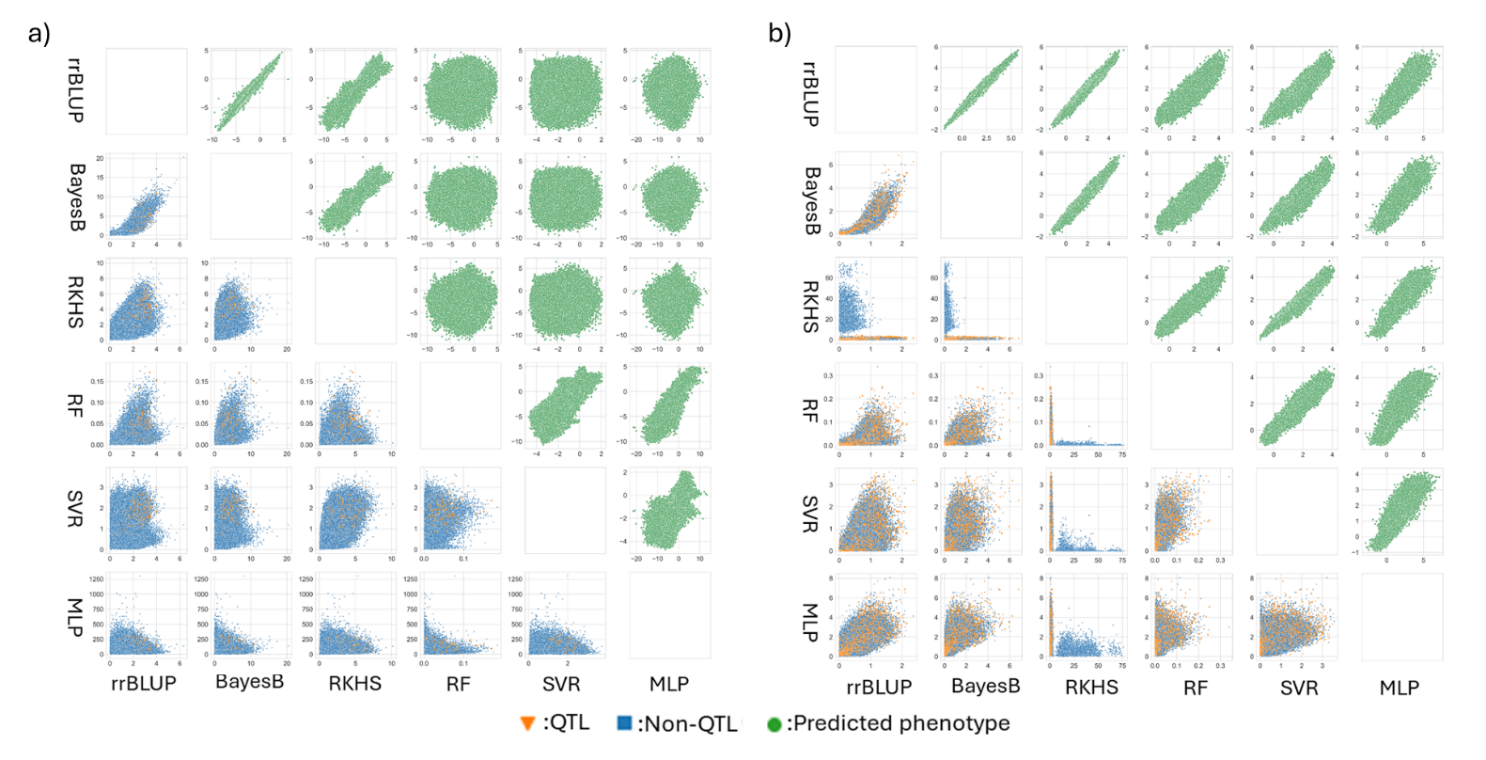


**Fig. S8:** Pairwise comparisons of the individual genomic prediction models (rrBLUP, BayesB, RKHS, RF, SVR and MLP) for the anthesis to silking interval (ASI) trait across all the prediction scenarios for the a) TeoNAM (7,500 prediction scenarios) and b) MaizeNAM (3,750 prediction scenarios) datasets. The prediction scenarios were generated by the combination of the 3 training-test ratios (0.8-0.2, 0.65-0.35 and 0.5-0.5), populations and sampling numbers. The genomic prediction models were compared at the level of predicted phenotypes (top right triangle) and genomic marker effects (the bottom left triangle). The green dots represent a pair of predicted phenotypes of RIL samples in the test sets for each prediction scenario. The blue squares and orange triangles represent a pair of extracted genomic marker effects from each genomic marker in each sample scenario that were identified as non-QTL and QTL markers, respectively, by Chen et al. (2019) for the TeoNAM dataset and Buckler et al. (2009) for the MaizeNAM dataset.

**
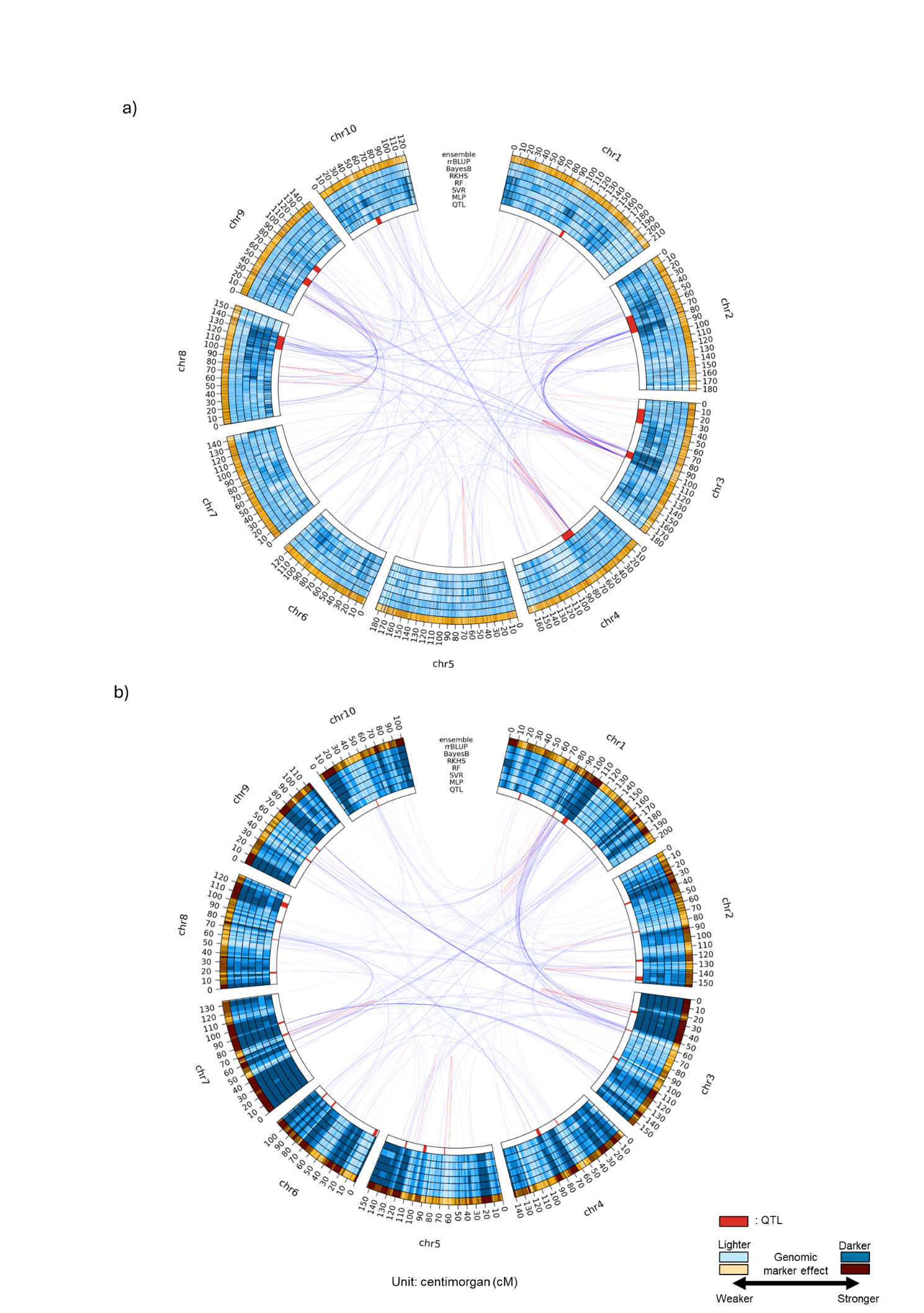
**

**Fig. S9:** Circos plots for the anthesis to silking interval (ASI) trait for the a) TeoNAM and b) MaizeNAM datasets. The innermost (QTL) ring shows the QTL gene regions estimated by Chen et al. (2019) for the TeoNAM dataset and by Buckler et al. (2009) for the MaizeNAM dataset. The blue rings (2nd to 7th) represent the genomic marker effects across the gene regions estimated by MLP, SVR, RF, RKHS, BayesB and rrBLUP, respectively. The outermost orange ring is the genomic marker effects for the ensemble-average model (ensemble). The numbers at the outermost ring represent genetic distance in centimorgans (cM). The darkness level of the blue and orange colors indicates the strength of the genomic marker effects, sectioned into 10 levels using the quantiles. Darker colors represent higher genomic marker effect levels. The red and blue lines between genome regions are the genomic marker interaction effects calculated by pairwise Shapley scores from RF (top 0.01%; red = within chromosome and blue = between chromosomes).


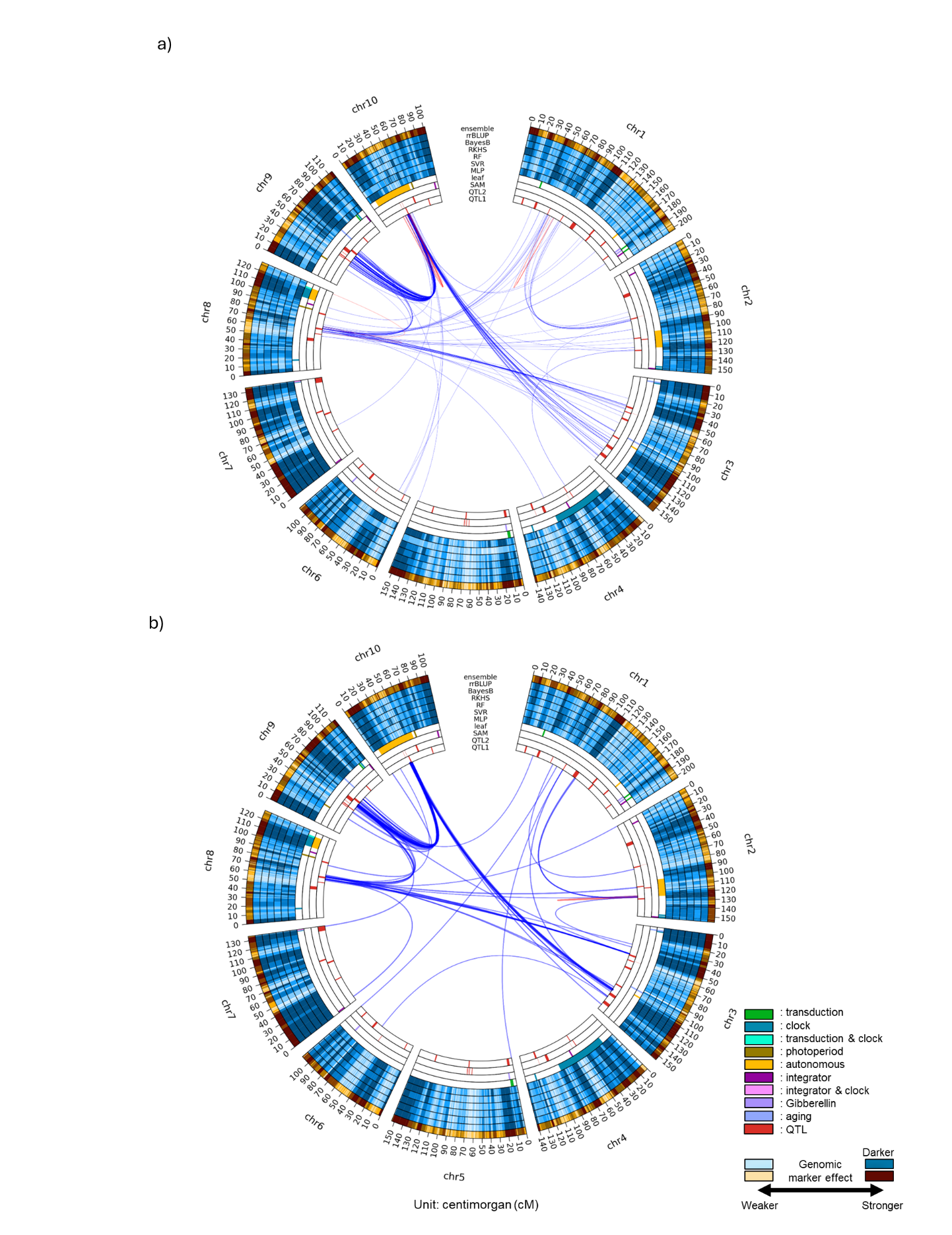


**Fig. S10:** Circos plots for the days to anthesis (DTA) trait for predicting the a) best linear unbiased estimates (BLUEs) and b) best linear unbiased predictions (BLUPs) in MaizeNAM datasets. The innermost (QTL 1) ring shows the QTL gene regions estimated by Chen et al. (2019) for the TeoNAM dataset and by Buckler et al. (2009) for the MaizeNAM dataset. The 2nd innermost ring (QTL 2) represents the QTL gene regions identified by Wisser et al. (2019). The 3rd and 4th innermost rings represent gene regulators that affect the shoot apical meristem (SAM) and leaf, respectively, identified by Dong et al. (2012). The blue rings (5th to 10th) represent the genomic marker effects across the gene regions estimated by MLP, SVR, RF, RKHS, BayesB and rrBLUP, respectively. The outermost orange ring is the genomic marker effects for the ensemble-average model (ensemble). The numbers at the outermost ring represent genetic distance in centimorgans (cM). The darkness level of the blue and orange colors indicates the strength of the genomic marker effects, sectioned into 10 levels using the quantiles. Darker colors represent higher genomic marker effect levels. The red and blue lines between genome regions are the genomic marker interaction effects calculated by pairwise Shapley scores from RF (top 0.01%; red = within chromosome and blue = between chromosomes).


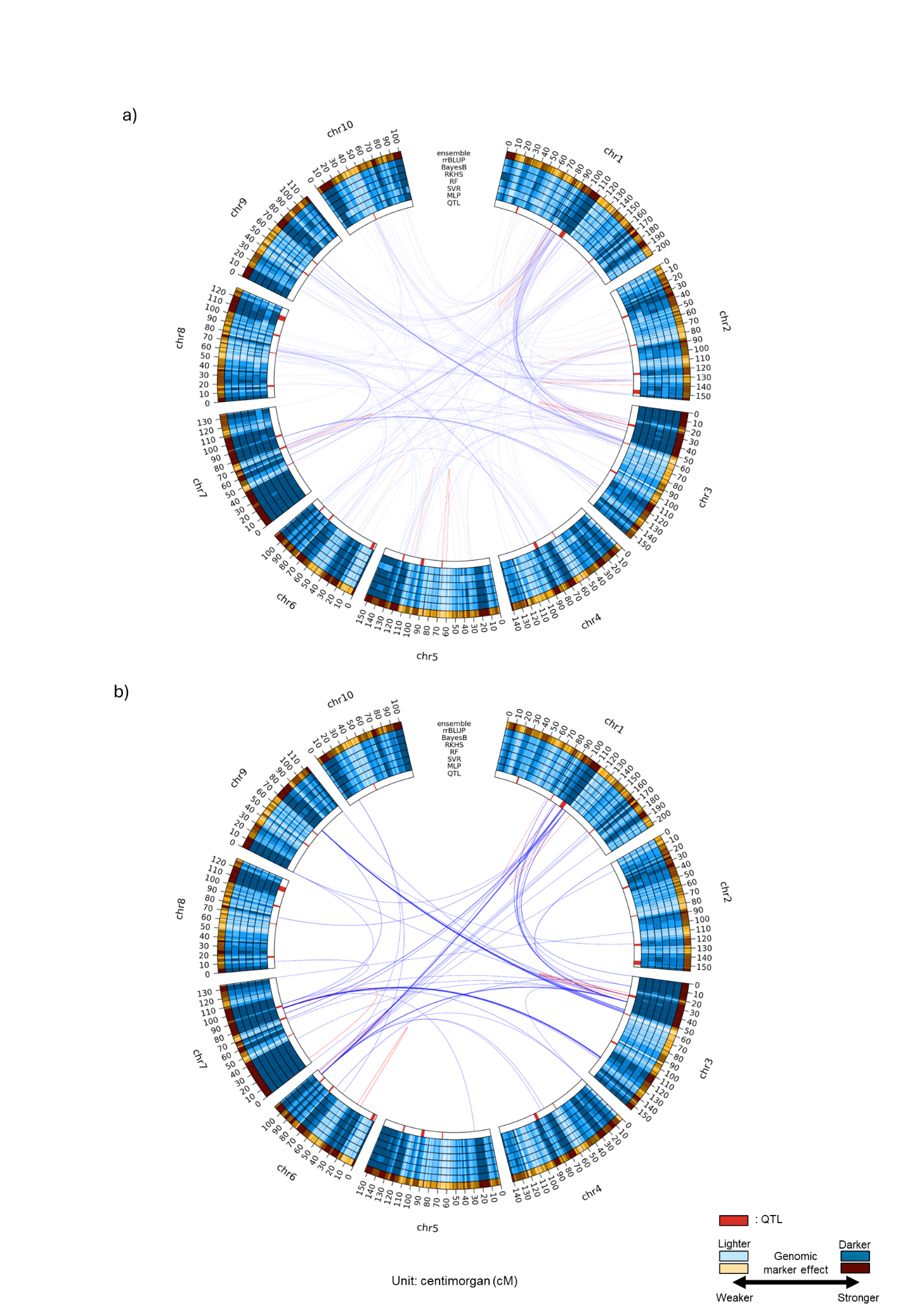


**Fig. S11:** Circos plots for the anthesis to silking interval (ASI) trait for predicting the a) best linear unbiased estimates (BLUEs) and b) best linear unbiased predictions (BLUPs) in MaizeNAM datasets. The innermost (QTL) ring shows the QTL gene regions estimated by Chen et al. (2019) for the TeoNAM dataset and by Buckler et al. (2009) for the MaizeNAM dataset. The blue rings (2nd to 7th) represent the genomic marker effects across the gene regions estimated by MLP, SVR, RF, RKHS, BayesB and rrBLUP, respectively. The outermost orange ring is the genomic marker effects for the ensemble-average model (ensemble). The numbers at the outermost ring represent genetic distance in centimorgans (cM). The darkness level of the blue and orange colors indicates the strength of the genomic marker effects, sectioned into 10 levels using the quantiles. Darker colors represent higher genomic marker effect levels. The red and blue lines between genome regions are the genomic marker interaction effects calculated by pairwise Shapley scores from RF (top 0.01%; red = within chromosome and blue = between chromosomes).
