# Supplementary figures and images for: Ensemble-based genomic prediction for maize flowering time improves prediction accuracy and reveals novel insights into trait genetic variation
Source: G3 (Bethesda). 2026 Apr 3;16(6):jkag090. doi: 10.1093/g3journal/jkag090 (PMC13232499; doi:10.1093/g3journal/jkag090)

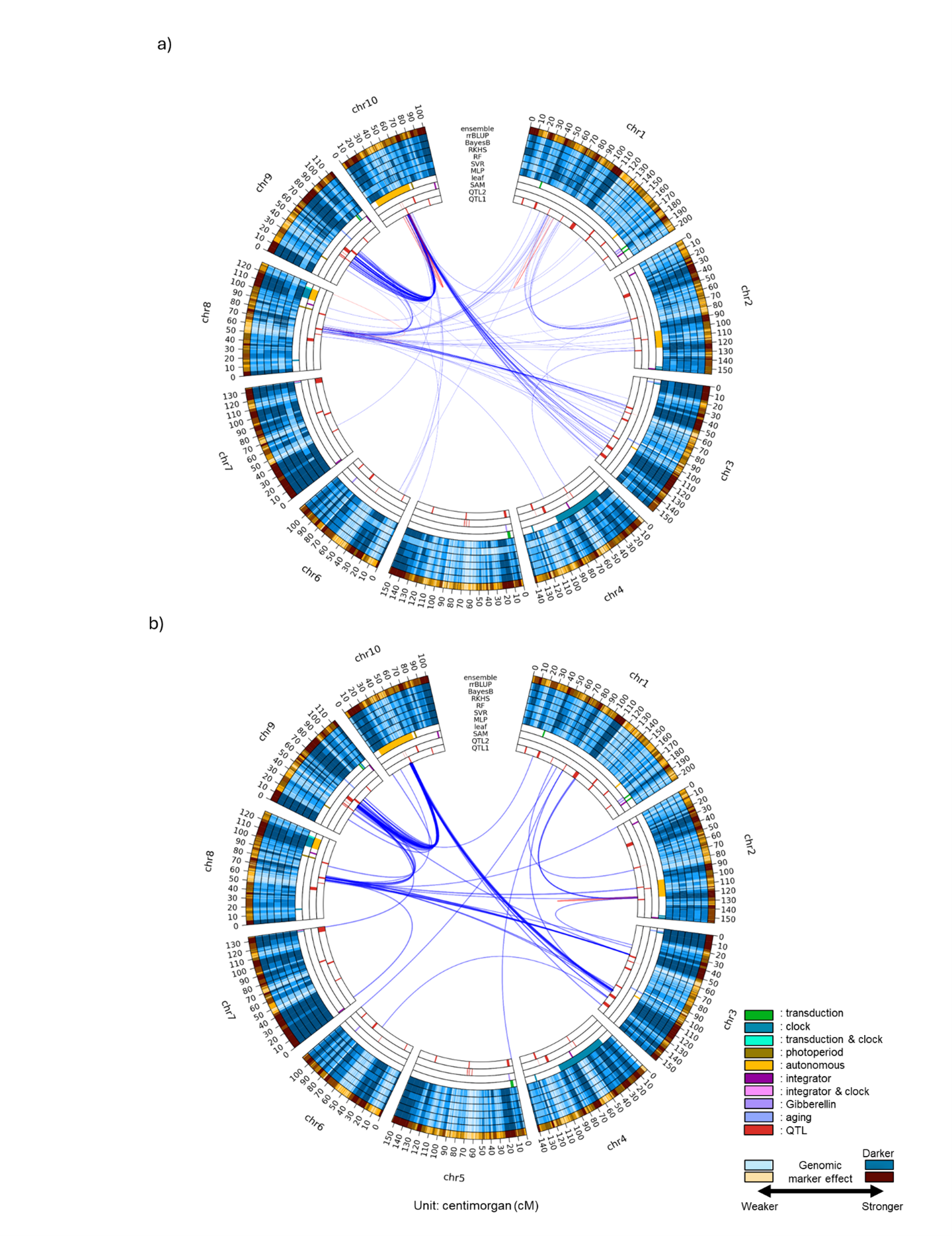

Supplement: jkag090_Supplementary_Data [file jkag090_supplementary_data.zip › Figure_S10_G3-2026-406684.png]

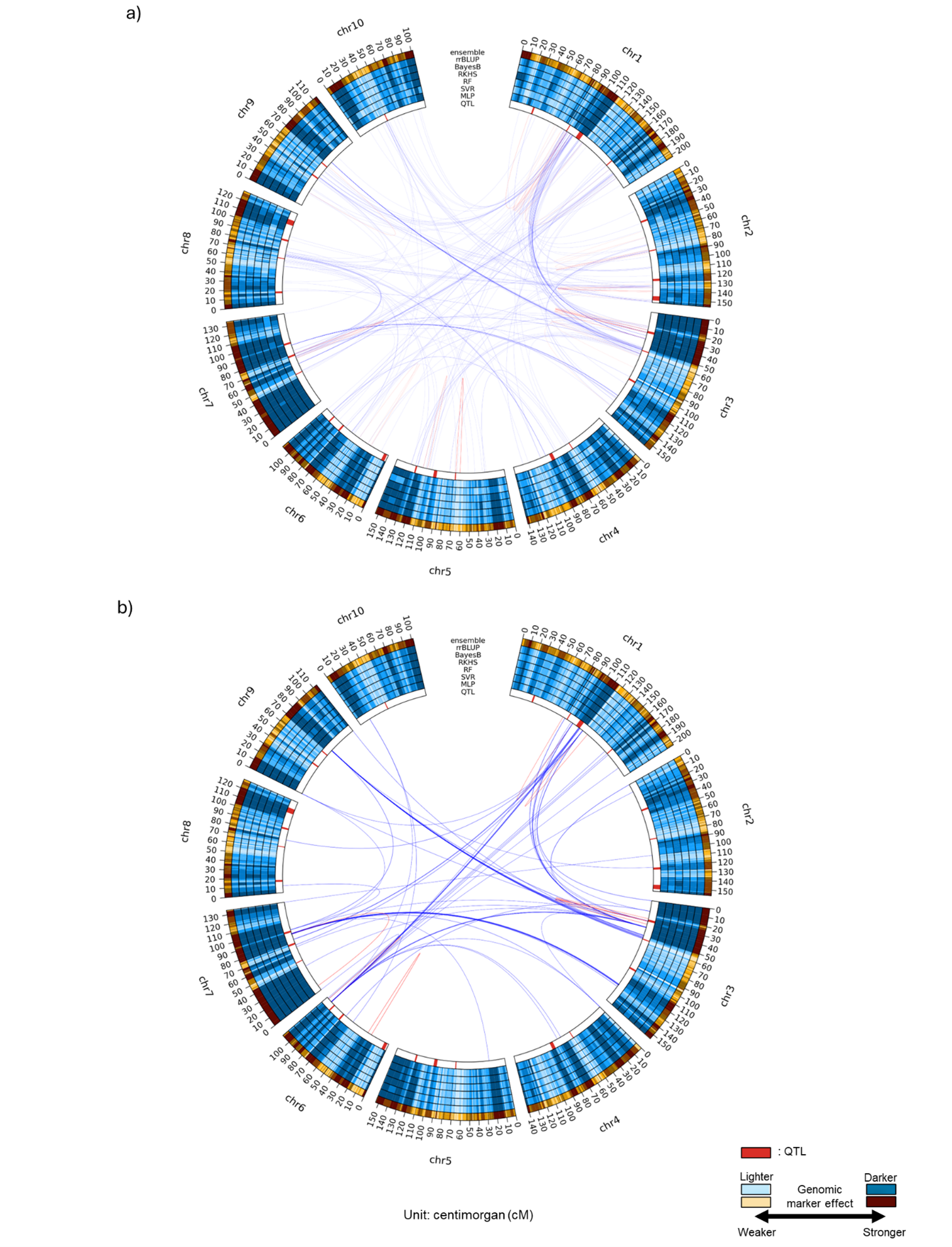

Supplement: jkag090_Supplementary_Data [file jkag090_supplementary_data.zip › Figure_S11_G3-2026-406684.png]

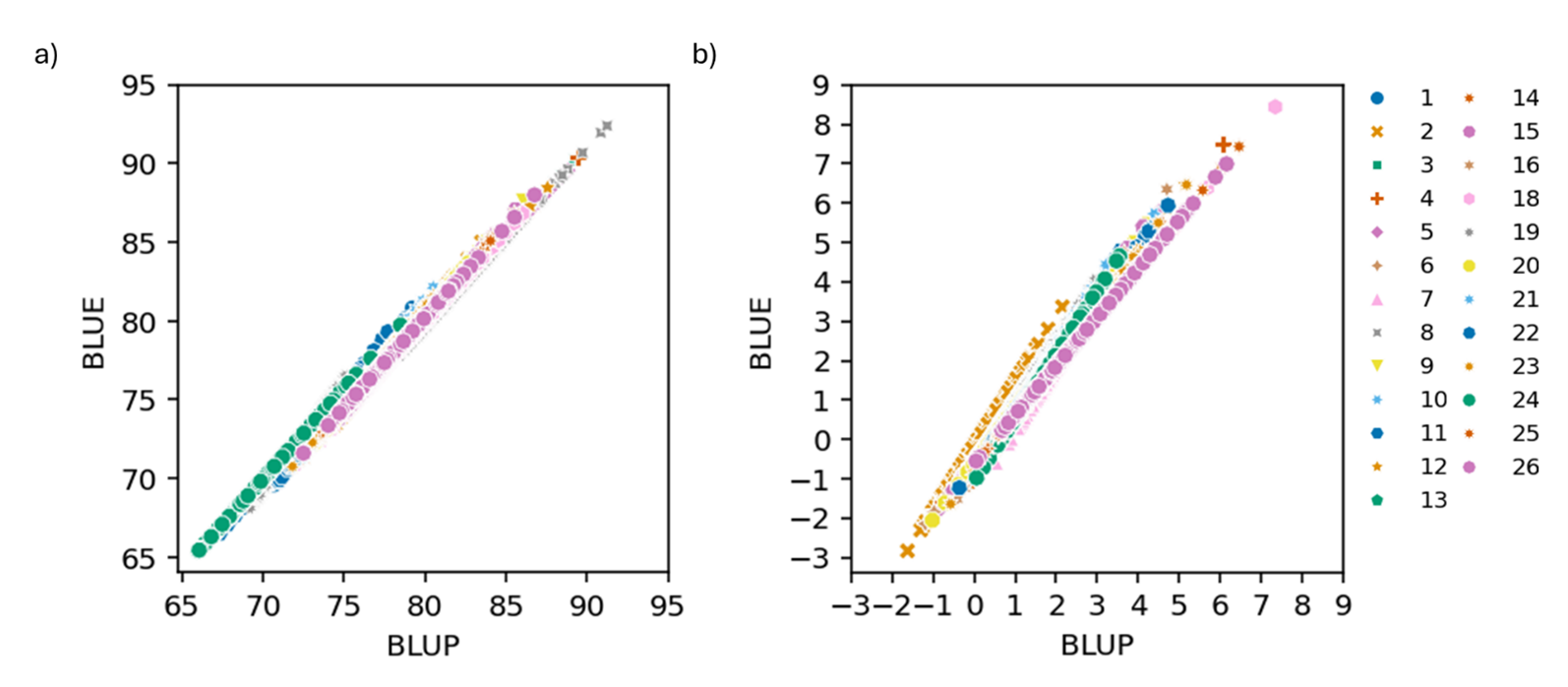

Supplement: jkag090_Supplementary_Data [file jkag090_supplementary_data.zip › Figure_S1_G3-2026-406684.png]

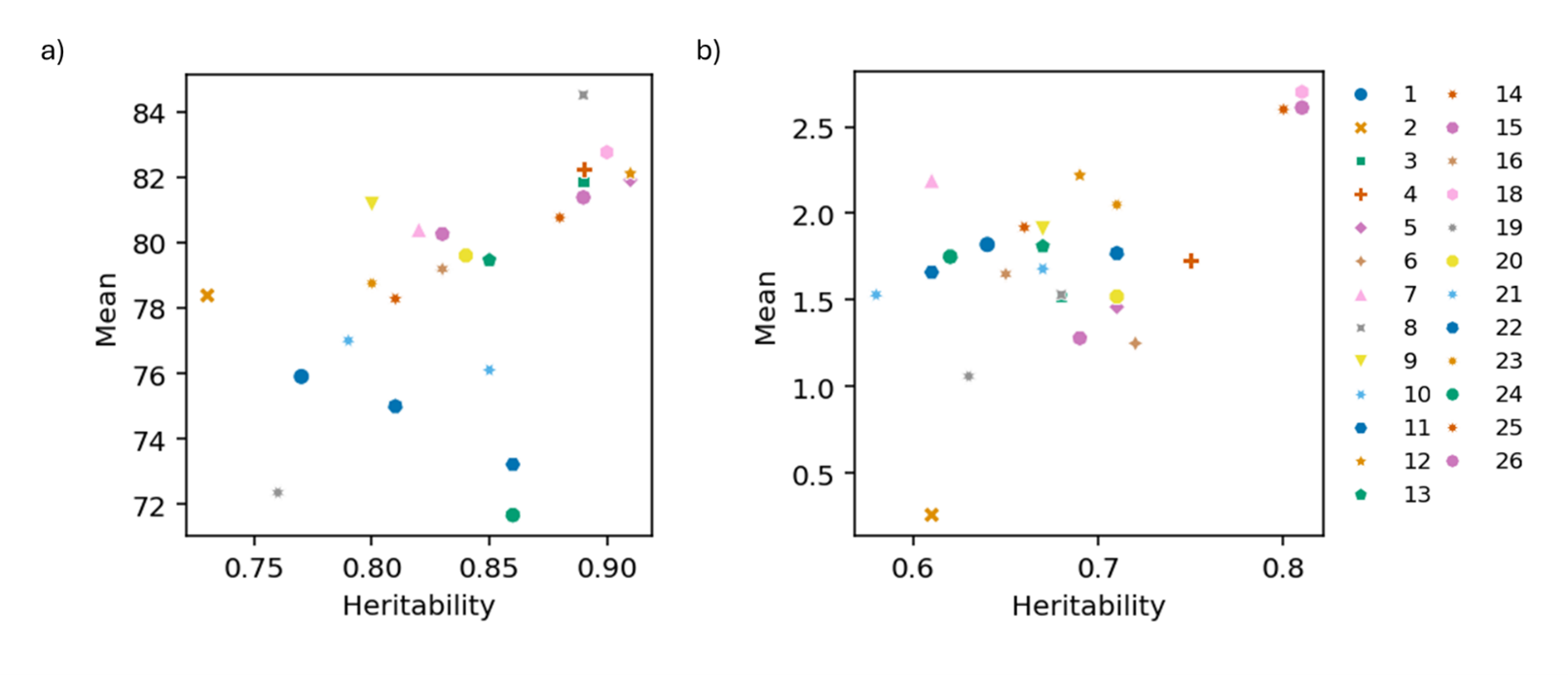

Supplement: jkag090_Supplementary_Data [file jkag090_supplementary_data.zip › Figure_S2_G3-2026-406684.png]

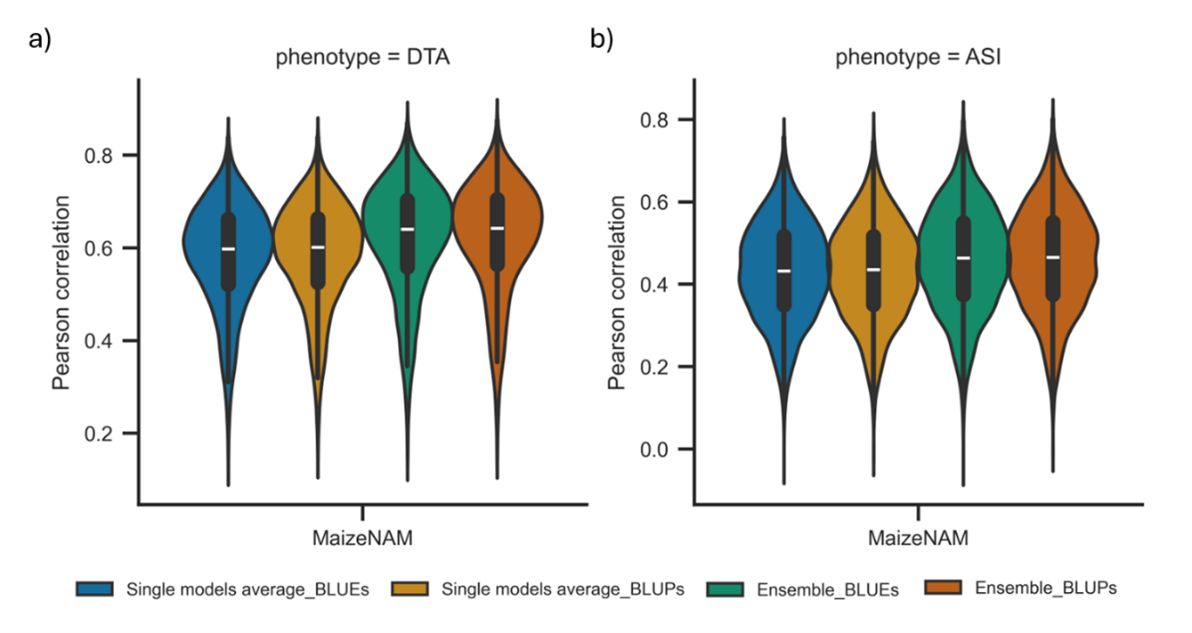

Supplement: jkag090_Supplementary_Data [file jkag090_supplementary_data.zip › Figure_S3_G3-2026-406684.png]

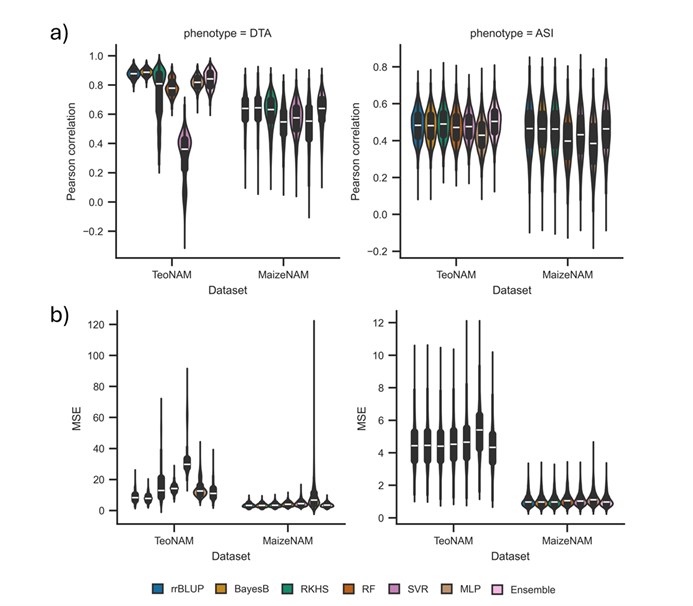

Supplement: jkag090_Supplementary_Data [file jkag090_supplementary_data.zip › Figure_S4_G3-2026-406684.png]

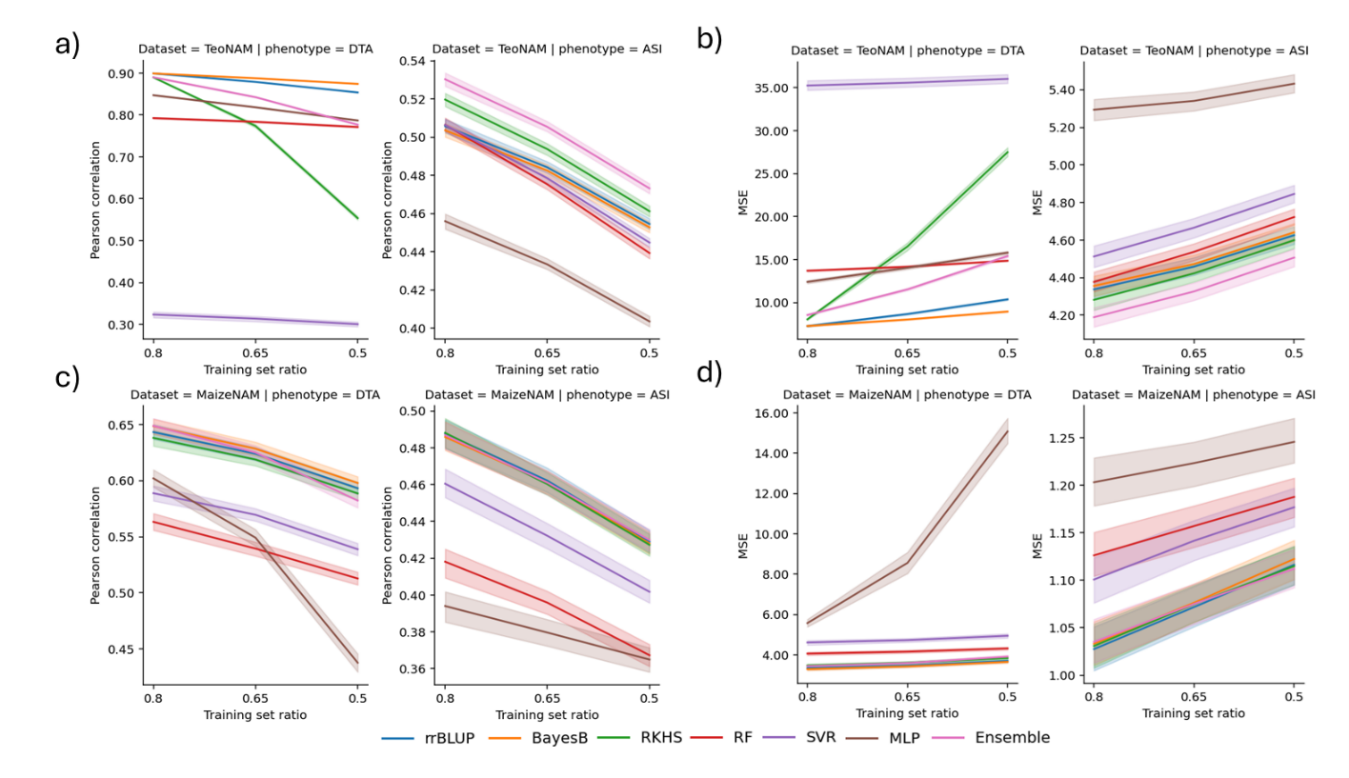

Supplement: jkag090_Supplementary_Data [file jkag090_supplementary_data.zip › Figure_S5_G3-2026-406684.png]

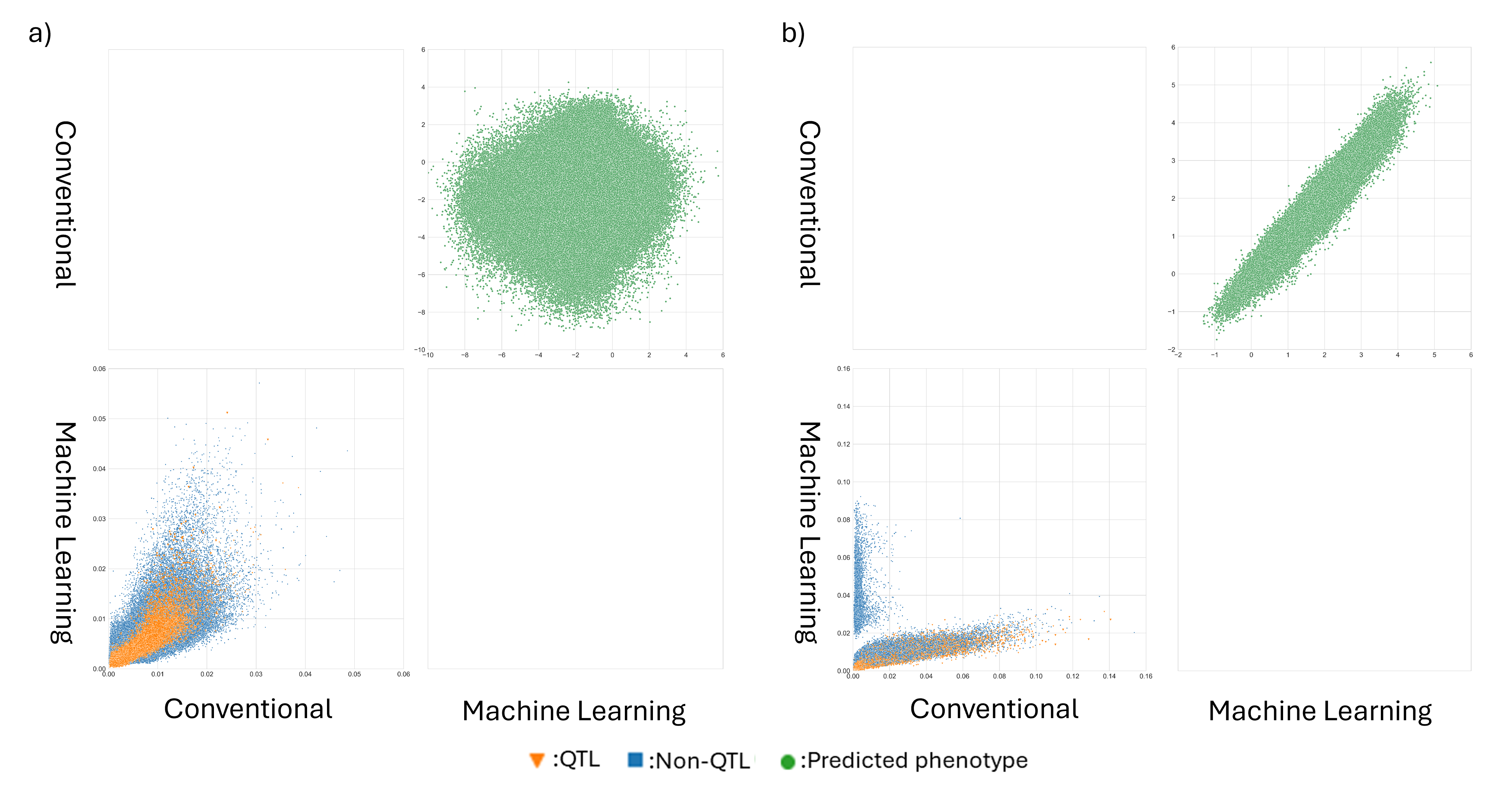

Supplement: jkag090_Supplementary_Data [file jkag090_supplementary_data.zip › Figure_S6_G3-2026-406684.png]

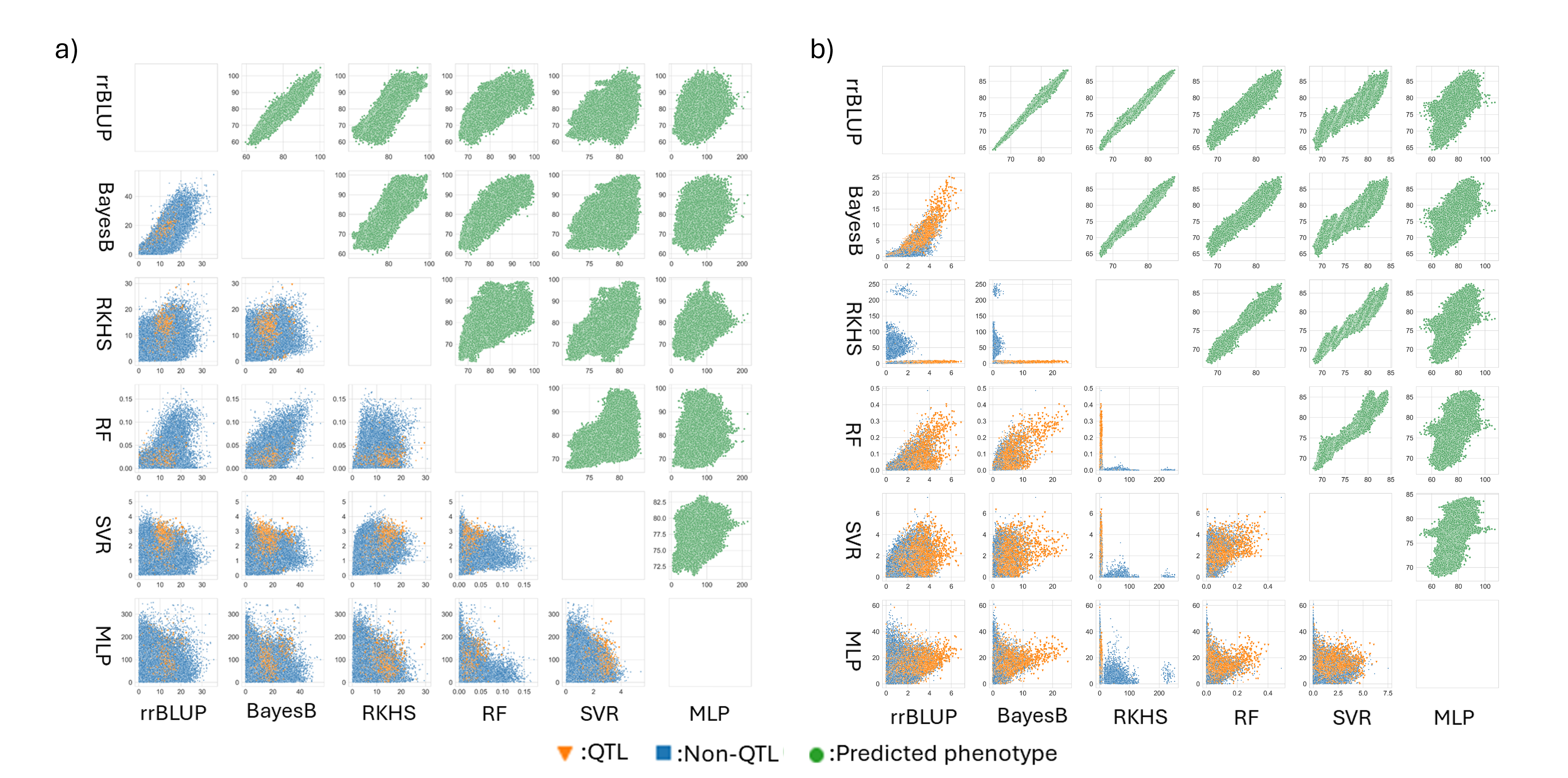

Supplement: jkag090_Supplementary_Data [file jkag090_supplementary_data.zip › Figure_S7_G3-2026-406684.png]

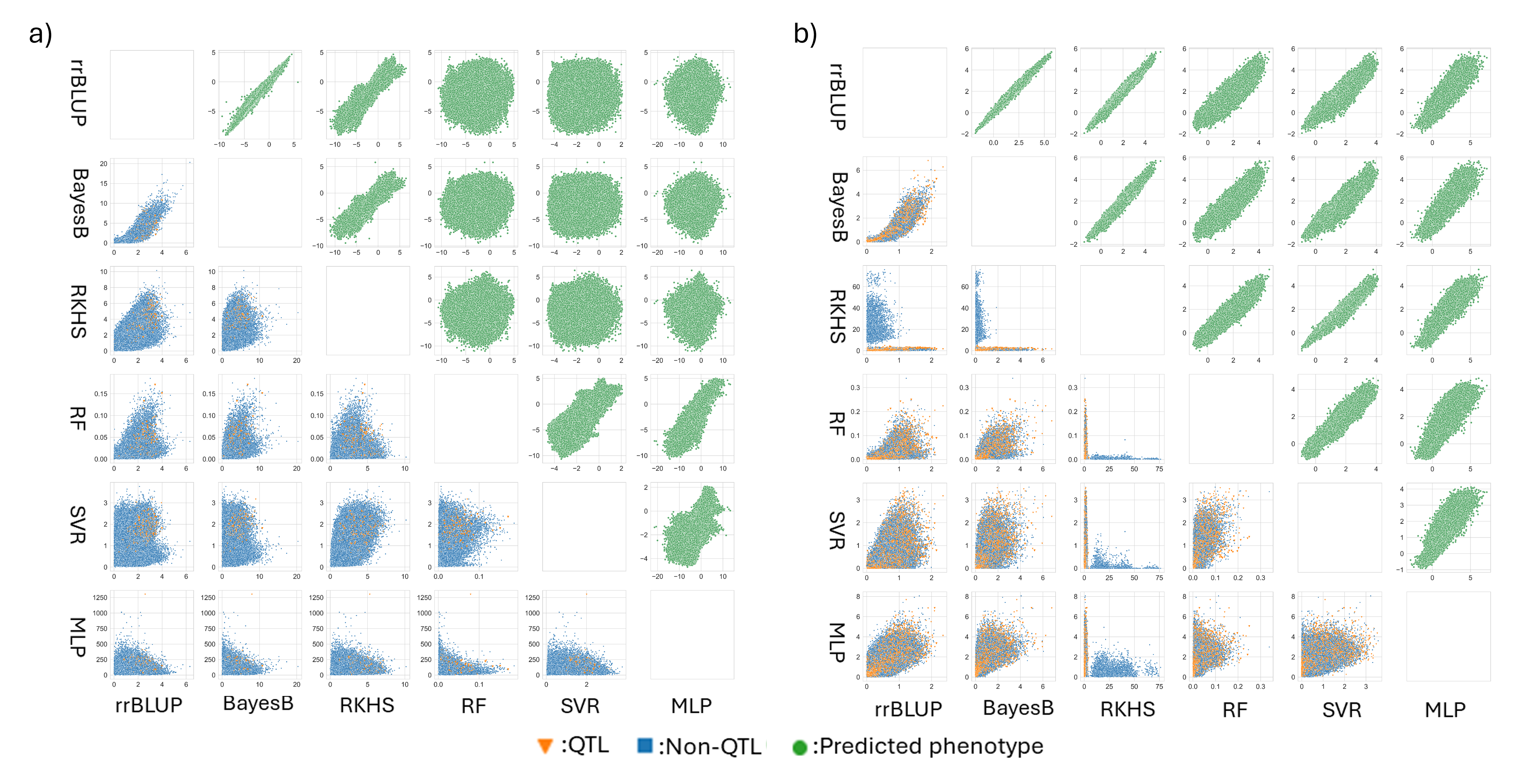

Supplement: jkag090_Supplementary_Data [file jkag090_supplementary_data.zip › Figure_S8_G3-2026-406684.png]

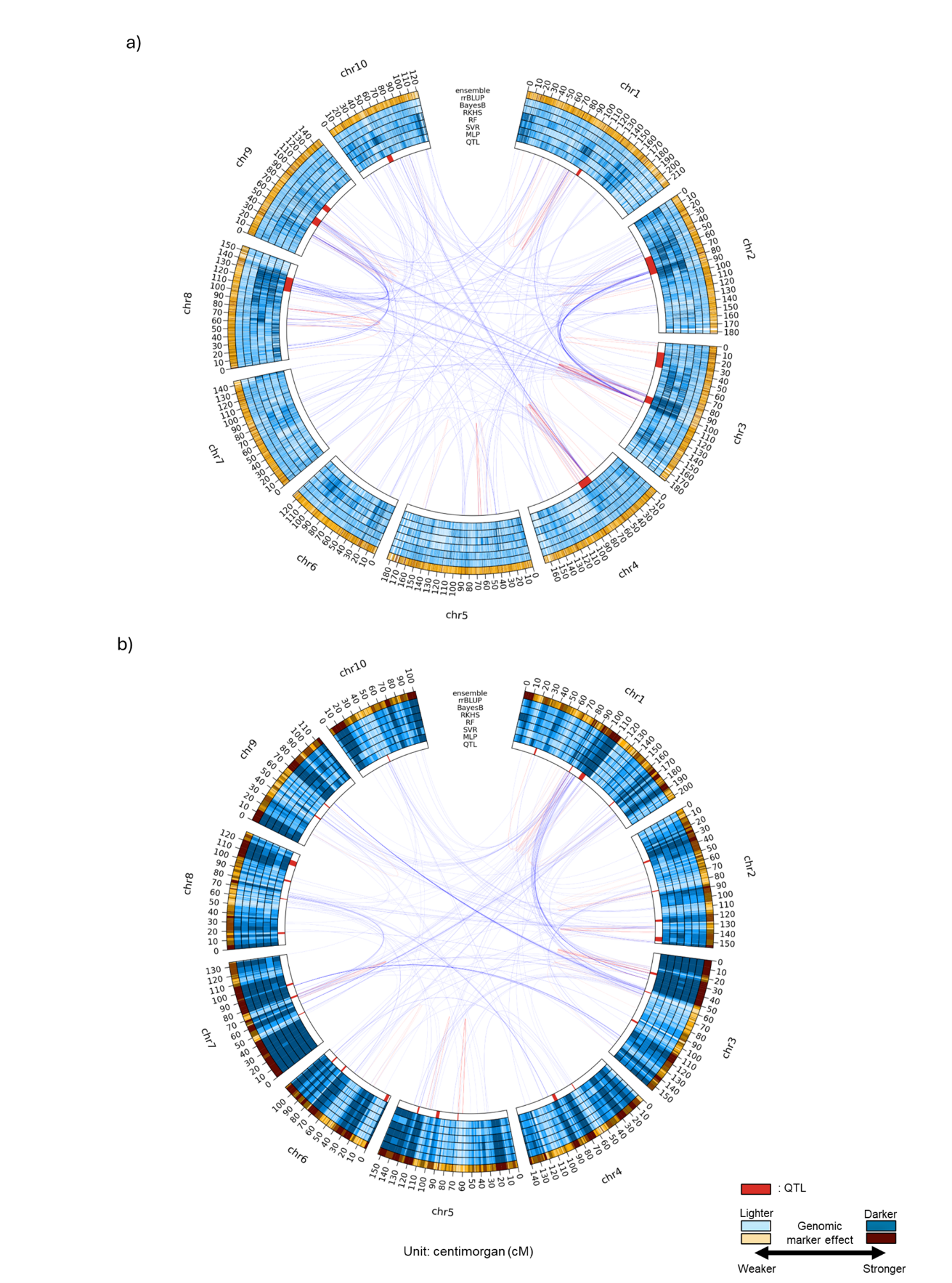

Supplement: jkag090_Supplementary_Data [file jkag090_supplementary_data.zip › Figure_S9_G3-2026-406684.png]
